# Feasibility and acceptability of psychosocial care for unsuccessful fertility treatment

**DOI:** 10.1111/hex.13598

**Published:** 2022-09-20

**Authors:** Mariana Sousa‐Leite, Mónica Fernandes, Salomé Reis, Raquel Costa, Bárbara Figueiredo, Sofia Gameiro

**Affiliations:** ^1^ Cardiff Fertility Studies Group, School of Psychology Cardiff University Cardiff UK; ^2^ Epidemiology Research Unit (EPI Unit), Institute of Public Health University of Porto Porto Portugal; ^3^ Laboratory for Integrative and Translational Research in Population Health (ITR) Porto Portugal; ^4^ Psychiatry and Mental Health Service University Hospital Centre of Porto (CHUP)/Northern Maternal and Child Centre (CMIN) Porto Portugal; ^5^ Department of Psychology University Hospital Centre of São João (CHUSJ) Porto Portugal; ^6^ Psychology Research Centre (CIPsi), School of Psychology University of Minho Braga Portugal

**Keywords:** acceptability and feasibility, preventive and early psychosocial care, psychosocial care, unsuccessful fertility treatment

## Abstract

**Introduction:**

Many people undergo fertility treatment to have biological children, but around four in ten patients complete all treatment cycles without having the children they desire. This triggers intense grief from which patients report taking on average 2 years to recover. Fertility guidelines and regulators stress the need to support patients through this process, but there is a scarcity of evaluated interventions to this end and evidence about when and how to offer care is lacking. This study explored patients' and healthcare professionals' (HCPs) experiences of and views about provision of psychosocial care (to patients facing unsuccessful fertility treatment, i.e., care provided by a mental health professional to address the emotional, cognitive, behavioural, relational and social needs that patients have at this stage of treatment).

**Methods:**

Five qualitative online focus groups were conducted with Portuguese participants: three with patients waiting to initiate or undergoing their last cycle of in vitro fertilization/intracytoplasmic sperm injection or having completed it within the last 2 months without achieving a pregnancy and two with HCPs working at fertility clinics. Focus groups were recorded and transcribed verbatim, and data were analysed with Framework Analysis.

**Results:**

Thirteen patients and nine HCPs participated. Analysis resulted in 1293 codes, systematically organized into 13 categories, 4 themes and 1 metatheme. The latter showed high consensus about the need for psychosocial care for unsuccessful treatment, but perceived challenges in its implementation. Themes reflected (1) consensual demand for psychosocial care at all stages of treatment but particularly at the end, (2) high perceived acceptability of integrating preventive care initiated during treatment with early psychosocial care only for those patients who experience unsuccessful treatment, (3) perceived challenges of implementing psychosocial care for unsuccessful treatment at clinics and (4) suggestions to promote its acceptability and feasibility.

**Conclusion:**

Patients and HCPs perceive that clinics should improve care provision across the whole treatment pathway and in particular for unsuccessful fertility treatment. Suggestions were made to inform future research focusing on the development and evaluation of psychosocial interventions to this end.

**Patient or Public Contribution:**

Patients and HCPs participated in the focus groups. Two HCPs also revised the manuscript.

## 1 INTRODUCTION

Parenthood is a universal goal shared by many people (77%–97%).^1^, ^2^ Over 9% of people worldwide face challenges to become parents (e.g., fertility problems or some life circumstances, such as being in a same‐sex relationship or being single).^3^, ^4^ In vitro fertilization/intracytoplasmic sperm injection (IVF/ICSI) are the recommended treatments used by many to achieve their parenthood goals.^5^ However, around 4 in every 10 people undergoing IVF/ICSI end all treatment cycles without achieving a live birth and need to adjust to a life without the children they desired.^6^ This study explored patients' and healthcare professionals' (HCPs') experiences of and views about provision of psychosocial care (to patients facing unsuccessful fertility treatment, i.e., care provided by a mental health professional to address the emotional, cognitive, behavioural, relational and social needs that patients have at this stage of treatment).^7^, ^8^

Unsuccessful fertility treatment, defined in this study as the last IVF/ICSI cycle reimbursed by the NHS being unsuccessful and no new cycles being attempted, triggers intense and prolonged grief.^9^, ^10^ National and international guidelines and regulators stress that it is the responsibility of fertility clinics to care for patients adjusting to this experience.^5^, ^7^, ^11^ However, there is a scarcity of evaluated psychosocial interventions to this end and a lack of evidence about when and how such interventions should be offered to patients. Patients report dissatisfaction with care at this stage of treatment, in particular, about being offered no closure from the clinic, no support resources and left to their own to face what they perceive as a catastrophic life event.^9^, ^10^

Preventive psychosocial care, delivered before unsuccessful treatment is experienced, can be helpful to provide information about common emotional reactions to validate and normalize experiences and minimize the impact of expected negative effects,^12^, ^13^ to foster hope by promoting self‐efficacy and agency in adversity^14^, ^15^ and to foster the therapeutic relationship, empowering patients to engage with timely support from their clinics after treatment ends.^16^ It can also promote patients' insight about the need for support, as many are overwhelmed by their grief reactions, or paradoxically, do not realize that they are grieving.^16^, ^17^ Offering this type of care is a recommended practice across several life‐threatening health contexts, when the futility of treatment is acknowledged and a shift towards discussion of the implications of this happens (e.g., end‐of‐life discussions).^18^ Such care was proved to be feasible, valued by patients and effective in sustaining their hope and quality of life during follow‐up periods.^19^, ^20^ However, evidence suggests that this is still not a common practice in fertility care.^21^, ^22^ One barrier may be that discussing possible adverse fertility outcomes is challenging for patients and HCPs alike because this triggers anxiety^23^, ^24^ and can reduce motivation for treatment.^24^, ^25^ However, the reality is that little is known about what are patients' and HCPs' views and preferences about preventive psychosocial care.

After unsuccessful treatment, early psychosocial care should target therapeutic goals known to promote psychosocial healthy adjustment in this context. The Three Tasks Model of Adjustment to Unmet Parenthood Goals (3TM) identified three therapeutic goals to be targeted: promoting acceptance of one's unmet parenthood goals (UPGs), that is, willingness to experience the loss and the emotions and thoughts that it triggers without struggle; facilitating meaning‐making, that is, construction of positive meanings related to one's UPGs and re‐evaluation of life values and priorities; and promoting the pursuit of meaningful alternative life goals.^17^ According to this model, promoting a favourable social context that supports patients in engaging with these therapeutic goals is also important to facilitate adjustment. Recent evidence supports the 3TM by showing positive associations between these three therapeutic mechanisms and mental health and well‐being.^26^

**Figure 1:**
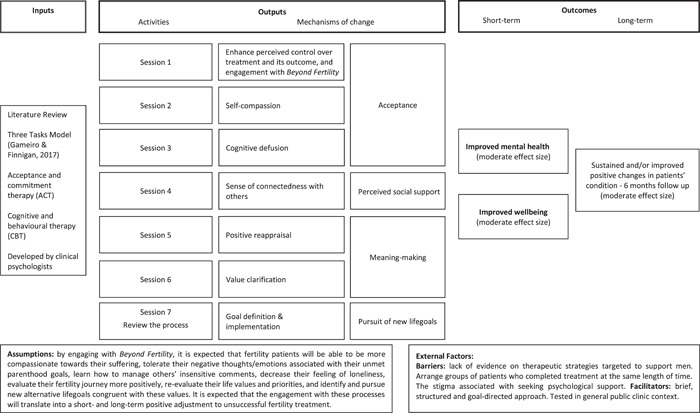
Logic model of the *Beyond Fertility* psychosocial intervention. Inputs represent the resources used to inform the development of the intervention. Outputs display the planned activities designed to target specific mechanisms of change (psychological processes). Outcomes represent the changes that are expected to be seen in real life after the planned activities are reached.

Following the Medical Research Council guidance,^27^, ^28^ the authors developed a psychosocial intervention for unsuccessful treatment: *Beyond Fertility* is a brief in‐person intervention, designed to support patients adjusting to unsuccessful fertility treatment. It is informed by the 3TM,^17^ applies contextual cognitive behavioural therapeutic principles, as these currently gather the most convincing high‐quality evidence of leading to effective psychosocial interventions,^29^, ^30^ and encompasses preventive (one individual/couple therapeutic session while patients prepare to initiate their last IVF/ICSI treatment cycle) and early (one individual/couple and five weekly group sessions starting 1–2 weeks after unsuccessful treatment) psychosocial care. Figure 1 shows the *Beyond Fertility* logic model, which is a visual representation of how this complex intervention works.^31^, ^32^

In sum, there is scarce evidence on how patients and HCPs experience provision of psychosocial care for unsuccessful treatment. Although offering preventive and early psychosocial care seems supported by research, it is unclear whether patients and HCPs perceive it as helpful and appropriate (i.e., acceptable) and possible to be implemented at fertility clinics (i.e., feasible). Research suggests that patients are willing to discuss the possibility of treatment cycles being unsuccessful if it helps them prepare for this eventuality,^24^ but discussing definite unsuccessful treatment may be far more challenging. Nonetheless, research also shows that patients lower their expectations when they progress through unsuccessful cycles^23^ and revise down their parenthood goals (and desire) as these become less achievable.^33^, ^34^ Therefore, it would be expected that patients become more willing to prepare for unsuccessful treatment as they perceive that they are more susceptible to it. In turn, HCPs report that discussing negative outcomes and managing patients' emotions in this context are major challenges in their clinical practice.^35^ Overall, evidence suggests low (HCPs) to moderate (patients) acceptability and provide no indication regarding the feasibility of implementing preventive and early psychosocial care for unsuccessful fertility treatment.

This study used Bowen's et al.,^36^ feasibility framework to investigate patients' and HCPs' acceptability and feasibility of psychosocial care for unsuccessful fertility treatment. Research questions were as follows:

1. What are patients' and HCPs' experiences and views of preventive and early psychosocial care for unsuccessful fertility treatment (i.e., acceptability)?
2. Is it feasible to implement psychosocial care for unsuccessful fertility treatment at fertility clinics (i.e., feasibility)?
3. Is a brief psychosocial intervention that integrates preventive (one session before the end of treatment) with early (six sessions after end of treatment, only for patients for whom treatment was unsuccessful) psychosocial care for unsuccessful treatment acceptable to patients and HCPs and feasible to be implemented at clinics?

## 2 METHODS

### 2.1 Participants

Participants were adult (aged 18 years or older) patients waiting to initiate or undergoing their last reimbursed IVF/ICSI treatment cycle (with own, fresh or cryopreserved, or donated gametes/embryos and with or without preimplantation genetic testing), or having completed their last cycle within the previous 2 months without achieving a pregnancy and HCPs (clinical psychologists [Psy], obstetricians and gynaecologists [OBS/GYN], embryologists [EMBs] and nurses) working at public Portuguese fertility clinics. Exclusion criteria were not being able to read or speak Portuguese.

### 2.2 Materials

#### 2.2.1 Demographic, professional and fertility treatment characteristics

Patients and HCPs were asked to state their age, gender, nationality, education and occupational status. Patients were additionally asked their area of residence, marital and parental status, current situation regarding fertility treatment and number of past IVF/ICSI cycles. HCPs were also asked to state their occupation, workplace and years of experience in fertility care.

#### 2.2.2 Focus group scripts

One semi‐structured script was developed following existing guidelines,^37^, ^38^ available in Supporting Information: Appendix S1. The script first introduced the topic of psychosocial care for unsuccessful fertility treatment and described the *Beyond Fertility* intervention. Open questions were informed by Bowen's^36^ framework and covered demand for implementation; practicalities: perceived needs that patients experience before initiating their last IVF/ICSI treatment cycle and after unsuccessful treatment and HCPs' perceived challenges in the provision of care to these patients; acceptability: perceptions and preferences towards care and the *Beyond Fertility* (presented to participants by means of its logic model); and implementation: perceived barriers and facilitators towards its execution. A final set of questions based on Mentimeter (interactive audience engagement platform) asked participants to describe, in one small sentence, the focus group and *Beyond Fertility*, to rate the extent to which they would recommend *Beyond Fertility* to a friend (patients only) or their patients (HCPs only) (from 1: *I would not recommend it at all* to 7: *I would totally recommend it*), how valuable it would be to implement it at clinics (*not at all*; *yes, maybe*; *yes, totally*) and the extent to which patients would engage/HCPs believed patients would engage with it over the seven sessions (from 1: *not at all* to 7: *will totally engage*).

### 2.3 Procedure

Consecutive female patients were contacted by phone, informed about the study and invited to participate with their partner. A convenience sample of HCPs was also invited via email. An information sheet and consent form, and the invitation link for the focus group session were sent to those willing to participate.

Five focus groups were carried out, separately with patients (September 2020 and January 2021) and HCPs (September 2020), to promote a safe and comfortable environment for participants to share their views.^37^ The focus groups were carried out via the zoom platform,^39^ audio‐recorded and transcribed verbatim. All groups were moderated by a clinical Psy and researcher (M. S.‐L.), and some assisted by another (S. G.). At the beginning of the focus group, its purposes and procedures were explained, and participants were alerted to the recording (as per consent) and informed about ground rules (e.g., confidentiality, absence of right or wrong questions, welcoming of all thoughts even if in opposite directions, freedom to ask additional questions). At the end, participants were provided with a link to access the short Mentimeter questions and submit their answers.

### 2.4 Data analysis

Descriptive statistics were used to characterize participants' background. Framework Analysis was used on the qualitative data to differentiate the views held by our different participant groups:^40^ patients (Pa), Psy and fertility specialists (Fs) (OBS/GYN, EMB, nurses). The verbatim transcripts were imported into NVivo software version 12.^41^ M. S.‐L. and S. G. familiarized themselves with the audio recordings and transcripts, and M. S.‐L. kept her data reflexions and impressions in a diary. Using an inductive approach, M. S.‐L. set codes (i.e., descriptive meaning labels) for each text segment of the first two transcripts. The research team (S. G., B. F., R. C. & M. S.‐L.) met several times to review the coding, and disagreements on interpretation were discussed until consensus was achieved. M. S.‐L. coded the following three transcripts applying the previous coding but allowing for new codes to emerge (also reviewed by the team). Connections and differences across the codes were analysed and systematically organized into categories representing similar ideas. A data matrix was created, with the categories in different rows, participant groups in columns and a summary of the codes with representative verbatim quotes in the cells (translated into English). (…) indicates that part of the quote was omitted as it did not add relevant information, and [text] represents clarifications added by the authors. The main categories were then organized into subthemes and main themes (i.e., interpretative descriptions of several categories describing interrelated ideas). A framework thematic map was created to illustrate the final matrix.

### 2.5 Ethical approval

The Ethics Committee of the São João Hospital Centre, Porto, Portugal (127/2020), and the School of Psychology of Cardiff University, Cardiff, UK (EC.21.05.18.6351), approved the study. The main ethical issue was that participation implied discussing challenging topics that could trigger negative emotions. Participants were informed that they could withdraw at any point without providing explanations and encouraged to contact the research team (accredited clinical Psy) if they had questions or concerns. Researchers were attentive to participants' reactions during the focus group (e.g., distress, discomfort) and available to contact them after the focus group, if needed.

## 3 RESULTS

### 3.1 Participants

Each focus group comprised 3–5 participants and lasted from 87 to 111 min (*M* = 99.00, SD = 9.08).

The final sample consisted of 10 women (27.03% participation rate), of whom three participated with the partner, and nine HCPs (42.86% participation rate). A total of 37 women were invited to participate, of whom 15 (6 with their partners) consented to participate, 10 refused to participate, mainly due to the emotional burden of treatment, work‐related activities and lack of time, and 12 stopped responding to the research team contacts. From those women who consented to participate (*n* = 15), one woman (with the partner) withdrew from the study at the beginning of the focus group (due to the emotional burden of the topic under discussion) and four (two of them with the partners) did not show up (due to unforeseen events, technological issues, unknown reasons). Of the twenty‐one HCPs invited, seven did not reply, one refused due to lack of interest, three withdrew from the study due to lack of time and unforeseen events and one did not show up due to unknown reasons.

Focus group composition and the characteristics of each participant's code (Pa, Psy, Fs) are presented in Table 1.

**Table 1:** Focus group composition and participants' characteristics and code

	FG 1	FG 2	FG 3	FG 4	FG 5
FG composition	5 Patients (2 couples)	3 Women (0 couples)	5 Patients (1 couple)	2 PSYCH, 1 GYN/OBS, 1 nurse	1 PSYCH, 2 GYN/OBS, 1 EMB, 1 nurse
FG duration (min)	95.12	87.10	98.13	111.43	103.23
Age *M* (SD)	39.20 (4.87)	37.67 (3.22)	38.80 (0.45)	48.33 (10.26)^a^	43.00 (4.90)
Gender	3 Women, 2 men	3 Women	4 Women, 1 man	4 Women	4 Women, 1 man
Education	2 Secondary school 3 BSc/BA/MSc	1 Secondary school 2 BSc/BA/MSc	4 BSc/BA/MSc 1 PhD	3 BSc/BA/MSc, 1 PhD	5 BSc/BA/MSc
Occupational status	5 Employed	1 Unemployed 2 Employed	5 Employed	4 Employed	5 Employed
Workplace				3 Public clinic 1 public and private clinic	3 Public clinic 2 public and private clinic
Work years in fertility care *M* (SD)				15.00 (2.83)	12.40 (6.27)
Area of residence	5 City	3 City	4 City, 1 village		
Marital status	4 Married or cohabiting 1 In a relationship without cohabiting	3 Married or cohabiting	5 Married or cohabiting		
Parental status	3 Childless, 2 with children	3 Childless	4 Childless, 1 with children		
Treatment point	4 Undergoing 1 Completed	1 Waiting to initiate 2 Undergoing	2 Waiting to initiate 3 Undergoing		
N° past IVF/ICSI cycles	3 With two cycles, 2 With three cycles	1 With no cycles, 1 with one cycle, 1 with more than three cycles	3 With two cycles 2 With three cycles		
Participant code	Pa2 (woman, childless, completed cycle), Pa3 (woman, childless, undergoing cycle), Pa6 (man, childless, undergoing), Pa9 (men, with children, undergoing), Pa13 (woman, with children, undergoing)	Pa4 and Pa12 (women, childless, undergoing), Pa8 (woman, childless, waiting to initiate cycle)	Pa1 and Pa10 (women, childless, undergoing), Pa5 (woman, with children, undergoing), Pa7 (woman, childless, waiting to initiate), Pa11 (man, childless, waiting to initiate)	Psy1 and Psy3 (PSYCH), Fs1 (OB/GYN), Fs2 (nurse)	Psy2 (PSYCH), Fs3 (nurse), Fs4 and Fs6 (OB/GYN), Fs5 (EMB)

Abbreviations: BSc/BA/MSc, Bachelor or Master; EMB, embryologist; FG, focus group; Fs, fertility specialist; GYN/OB, obstetrician and gynaecologist; *M*, mean; Pa, patient; PhD, Philosophiæ Doctor; Psy, psychologist; PSYCH, psychologist; SD, standard deviation.

^a^ One participant did not report on their age.

### 3.2 Data generation

**Figure 2:**
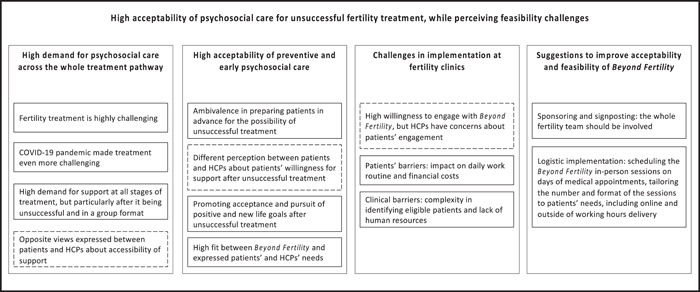
Framework thematic map. Thirteen categories of codes grouped into four themes and one metatheme. Continuous lines represent consensus between patients and healthcare professionals (HCPs) and dashed lines represent some level of disagreement.

Framework Analysis yielded 1293 different codes, which were systematically organized into 13 categories, grouped into 4 themes and 1 metatheme. Figure 2 shows the framework thematic map. Supporting Information: Appendix S2, presents the final framework matrix.

The metatheme *High acceptability of psychosocial care for unsuccessful fertility treatment, while perceiving feasibility challenges*, reflected a high demand for psychosocial care at all stages of treatment, but in particular in the aftermath of unsuccessful treatment. Overall, participants reported positive views and perceived benefits in the provision of preventive and early psychosocial care for unsuccessful treatment but had concerns about if and how to forewarn patients for this possibility. *Beyond Fertility* was perceived to meet patients' needs. Challenges and suggestions for its successful implementation were highlighted.

#### 3.2.1 High demand for psychosocial care across the whole treatment pathway

All participants reported that fertility treatment is extremely challenging at all treatment stages, in particular, during the last cycle. It has a negative impact on individual and relational well‐being and leads to significant mental health problems for a minority of patients.

(…) it has not been an easy process at all, quite the contrary, each treatment is increasingly difficult, psychologically it has been a drastic shock (…) but I think in these situations [unsuccessful cycle attempts] we [patients] are all in it together, aren't we!? (Pa1)

Over time, patients seemed to find positive coping strategies to tackle treatment burden, but unsuccessful treatment triggered a ‘grieving process’ (Psy2), with patients feeling that they were ‘in a riot’ (Pa2). The COVID‐19 pandemic was perceived as ‘one more thing to make me anxious’ (Pa8), mainly due to increased waiting periods and uncertainty about access and time of future treatment cycles.

Participants' perceptions of treatment as being highly challenging seemed to drive a consensual demand for psychosocial care at all stages of the process and particularly after unsuccessful treatment and for those with fewer emotional and relational resources. All participants thought that support should be offered to both members of the couple, and two highlighted that it should be provided by a mental health professional with expertise in fertility care.

most importantly, when we finish this process [fertility treatment] and things do not go well, and we no longer have a connection [with patients], I think psychological care is essential. (Fs5)

While patients and HCPs agreed on the need for psychosocial care, both expressed different views about its accessibility. HCPs reported that patients could ask for support at any time, but that specialists only refer those with ‘significant emotional distress, which is somehow interfering with the treatments being carried out by the medical team’ (Psy1). Only one couple reported being offered psychosocial care and was dissatisfied with its provision. All other patients stated that ‘as Pa4 said a while ago [“no one has ever signposted me, neither in private [clinics] nor here”], no one asked me anything, no one asked me: do you need it?’ (Pa8), stating unawareness regarding available psychosocial care. Patients and HCPs expressed a preference for group‐based psychosocial care, in which patients have the opportunity to share experiences with people living in similar circumstances, learn from others' experiences and feel less isolated. Such interventions were not available at clinics, although one Psy reported that it was something that they ‘have been thinking about for many years’ (Psy2).

Yes, I think sharing is important as well, and realising we are not alone, we are not the only ones going through the same situation. Yes, I fully agree. (Pa13)

#### 3.2.2 High acceptability of preventive and early psychosocial care

Both patients and HCPs stated that supporting patients adjusting to unsuccessful treatment is highly needed and were willing to engage with it before and after treatment. However, HCPs perceived that most patients are not willing to receive it immediately after treatment being unsuccessful as they are too overwhelmed with grief: ‘very frustrated, very angry, very discouraged’ (Psy1), and that in general, the male partner is less willing to engage, as ‘the tendency is for women to come alone’ (Psy2). Most HCPs suggested that psychosocial care should only be provided 1–2 months after unsuccessful treatment, once intense grief reactions have subsided, but agreed that if provided much later, patients could feel ‘helpless’ (Psy1) or ‘no longer need it’ (Fs1), as they would have already ‘moved on’ (Fs1). In contrast, all patients claimed that they were willing to receive such support at any stage of treatment, in any format and preferable immediately to 2 weeks after the end of unsuccessful treatment.

Preventive care (before the end of treatment) to prepare patients for the possibility of unsuccessful treatment was considered important and beneficial by both patients and HCPs, but different views on how to approach it were reported. All HCPs stated that it is important to manage patients' expectations in a balanced and realistic way, but reported using different approaches to do it (e.g., fostering hope vs. contemplating failure). Patients agreed that preventive care was needed, but reported that the clinic setting and communication from HCPs, in particular from medical doctors, were sometimes insensitive (‘the way they told me: look, your treatment was negative, now you go home, wait, and in a year we call you again. And that's it’, Pa1) and did not provide strategies to help them manage expectations and prepare for treatment being unsuccessful: mostly focused on positive outcomes, inflation of the probability of success, lack of explanations for the treatment plan and unsuccessful cycles/treatment.

the doctor when, when she came to me at the end, saying: ‐yeah, it was very good, we got 16 oocytes, wonderful. We were left with an expectation, huh, huge, huge (…), and sometimes it doesn't mean that. (Pa1)

I agree with Pa5 and P10, we really need a lot of support to manage our expectations. (Pa1)

Overall, most patients and HCPs agreed that preparation for unsuccessful treatment is imperative, in particular, at the later stages of treatment. It should be based on an empathic approach, focused on positives and tailored to each patient's individual willingness and preferences, always reassuring them that psychosocial care is available at any point. Suggested topics to be addressed included coping strategies to manage distressful emotions and thoughts, common adjustment experiences, how couples can rethink their future together and alternative life paths beyond (biological) parenthood.

a very big fear (‥) what if, if we never make it, huh!? What's going to happen to us [as a couple], you know!? (…) maybe it is important for you and your partner to talk about it, you have this fear, but he has it as well, isn't he? (Pa3)

‘of course’. (Pa2)

On the other hand, three patients and one Psy disagreed, claiming that there is no point in preparing patients for something that may not happen, highlighting that it could interfere with patients' engagement with treatment.

‘I don't know if it would not be stressful, at that stage, before treatment, to be contemplating this possibility when the door is not yet closed’ (Pa6).

‘No, I don't think so either. I don't think so, it's not time, it's time for us to have all our strength up, with our good mood, our optimism, our hope’. (Pa2)

According to patients and HCPs, psychosocial care in the aftermath of unsuccessful treatment should help patients to accept and normalize negative emotions, feelings and thoughts, manage difficult social interactions, accept their inability to have biological children, increase focus on the positive aspects of life and explore other goals beyond biological parenthood.

(…) we need to know how to deal with each other as a couple after this, because it's been a lifetime thinking that this will happen sooner or later, and suddenly there's that, no, it's just the two of us. (Pa2)

I think this intervention has to show that in life people have to have several interests, because unfortunately people do not always succeed in all areas, but they [patients]) have to focus on family, friends, activities that people like to do, don't they!? at work, and so there's actually other things besides that. (Fs1)

*Beyond Fertility* was perceived by patients and HCPs as covering a currently unmet need in reproductive mental healthcare. Patients and HCPs agreed that its logic model is holistic and tailored to meet patients' mental health needs and that overall, its therapeutic objectives (i.e., mechanisms of change) address the needs experienced after unsuccessful treatment. All were highly willing to engage with it.

indeed, the balance is achieved precisely with these four (Beyond Fertility mechanisms of change) [laughs]. If all are achieved, we get there. (Fs5)

Participants appreciated that *Beyond Fertility* included both individual/couple and group sessions, since these would allow them to work on different goals, making both formats helpful, with particular preference towards group sessions.

I agree with Pa6, I think both the individual and the group [sessions] are important, because they have different goals, I think, for sure, don't they!? (Pa3)

#### 3.2.3 Challenges in implementation at fertility clinics

HCPs considered that it can be challenging to identify patients starting their last cycle, as many may undergo additional cycles in the private sector or with gametes/embryos donation. In addition, long waiting lists make it difficult to anticipate when patients will start the cycle and schedule the first session.

(…) But then (after unsuccessful treatment) we can offer alternative options, and therefore the alternatives can go through, as already mentioned here, gametes or embryos donation. (Fs1)

HCPs also mentioned that it may be difficult to have both members of the couple, especially the male partner, engaged over time, and that some patients may not participate in group sessions, a view supported by patients. Patients and HCPs agreed that in‐person sessions are difficult to manage due to travelling costs, time and work absences, including the need to disclose infertility at work and the associated stigma. HCPs also noted the lack of human resources, in particular, mental health professionals, with a tendency to worsen over time. Patients echoed this perception, as they feel that the public sector is overloaded, even more during the COVID‐19 pandemic, and the private sector is costly.

It's just that I should not feel shame, should I!? But that's what I feel sometimes, honestly. Apart from feeling very exposed. One thing is to say: look, I will be absent [from work], I have a medical appointment. And they don't even ask me [why] (…) but the documentation goes through several hands, and the fact that it has reproductive medicine there. (Pa4)

Even more if it says psychology of reproductive medicine. (Pa8)

#### 3.2.4 Suggestions to improve acceptability and feasibility of beyond fertility

Patients wanted to be informed about *Beyond Fertility* by a member of staff they feel comfortable with, specifically the nurses, as patients feel—‘there is a link, a stronger connection (…)’ (Pa7),—‘I agree with, with Pa7. I think the nurses end up giving us more psychological support’ (Pa1), but also found it acceptable to receive a phone call from a Psy. HCPs referred *Beyond Fertility* should be introduced by the medical doctors in one of their appointments, followed by a telephone contact from the Psy. Both patients and HCPs thought it important to tailor the number of individual sessions after the end of treatment to patients' needs, as some patients might need more than two sessions before moving on to the group sessions. All agreed that patients should be able to choose between in‐person and online sessions, to avoid circumstantial barriers (e.g., travelling costs and time). If patients preferred in‐person sessions, agreement was these should be scheduled on medical appointment days. Although all participants agreed that sessions should be conducted outside of working hours to circumvent work‐related constraints, four HCPs claimed that it would not be feasible as ‘the people delivering the intervention end up working outside of working hours’ (Fs1).

the fact that there are many sessions, if they are in‐person, I don't know if couples will easily accept them. I think doing the group sessions outside of working hours, via Zoom, will increase acceptance. (Fs1)

Yes, yes (…) I think the use of technologies here can be an asset. (Psy1)

because in that way (online and outside of working hours) nobody knows where I am, I don't have to miss work, I don't have to travel, I don't have work piled up… All this weighs, doesn't it!? (Pa4)

#### 3.2.5 Mentimeter results

All participants answered the online questions. Patients and HCPs considered the focus group discussion ‘very interesting’ (*n* = 6) *and/or* ‘productive’ (*n* = 6) and an opportunity to ‘share experiences’ (*n* = 5). Two patients added that sharing their experiences with other patients going through the same experience was ‘good’ and ‘made a difference’ to them. Patients considered *Beyond Fertility* ‘very useful’ and/or ‘an essential help to support many couples’ (*n* = 7), and HCPs considered it ‘novel’ (*n* = 3), ‘highly relevant’ or ‘needed’ (*n* = 4) and an ‘added value for couples’ (*n* = 2). Patients were highly willing to recommend *Beyond Fertility* to a friend (*M* = 6.50, SD = 0.87) and HCPs to their patients (*M* = 6.65, SD = 0.21). All participants agreed that it would be an asset to implement *Beyond Fertility* in clinics (patients: *Yes, totally*: *n* = 10, 100%; HCPs: *Yes, totally*: n = 7, 77.78%, *Yes maybe*: *n* = 2, 22.22%). Finally, patients were highly willing to engage with *Beyond Fertility* over the seven sessions (*M* = 6.10, SD = 0.53) but HCPs were not so sure about patients' engagement (*M* = 3.60, SD = 0.28).

## 4 DISCUSSION

Fertility patients and HCPs require and accept provision of psychosocial care across the whole treatment pathway and in particular for unsuccessful treatment. Implementing psychosocial care for unsuccessful treatment at fertility clinics is desirable and seems possible. Interventions that integrate preventive care, offered in an empathic and hopeful way towards the end of the treatment pathway to promote validation and normalization of emotional reactions and coping skills, and early psychosocial care guided by the 3TM are likely to be accepted by patients and HCPs. How psychosocial care is implemented in clinics is critical for acceptability and feasibility. Sponsorship from the whole fertility team, signposting from familiar nurses and medical doctors before contact with a mental health professional, online delivery options and some level of tailoring to patient profile seem to be important requirements.

Results highlight the contrast between patients' high demand but perceived low accessibility of psychosocial care for unsuccessful treatment. This gap in care provision has been previously identified^42^ and is associated with patients' frustration and dissatisfaction towards their clinics.^9^, ^10^ Our results suggest that clinics' inability to meet patients' needs may result from lack of human resources, HCPs' perceptions that patients are not willing to engage with support and concerns that it may interfere with patients' ability to continue with treatment. Participants were proactive in suggesting approaches to provision of psychosocial care for unsuccessful treatment. These highlighted that care provision should be an endeavour of the clinic with involvement from all staff and that it requires skills in empathic communication, expectations management, validation and normalization of reactions to treatment events and fostering hope in adversity.

If preventive psychosocial care for unsuccessful treatment is to be implemented at clinics, it needs to be tailored to patient profile (e.g., poor prognosis) or offered towards the end of the treatment pathway (e.g., after two unsuccessful cycles), at a time patients are more willing to disengage from treatment and contemplate alternative life paths and goals.^25^, ^43^ Clear signposting should be done by a trusted member of staff in combination with a phone call from the mental health professional delivering such support.

Addressing HCPs' concerns about negative impacts of preventive care and misconceptions that it is not desired by patients will also be crucial to implementation. Similar cultural shifts have enabled provision of preventive care in other life‐threatening health contexts (e.g., end‐of‐life conversations) with positive outcomes for patients. Crucial to this shift was mapping patients' preferences in this regard, much like in the current study. Furthermore, HCPs may also need to be supported in developing the skillset needed to discuss the possibility of treatment being unsuccessful. Fertility bespoke training on how to share bad news^44^, ^45^ and empathic communication^46^ can provide HCPs with opportunities to develop some of these skills.

Early psychosocial care grounded on the 3TM^17^ is considered useful and adequate to address most of the needs that patients experience in the aftermath of unsuccessful treatment. An additional therapeutic goal to consider when working with couples is the promotion of intercouple communication and exploration of alternative joint futures, which requires a dyadic approach to care provision. Early psychosocial care after unsuccessful treatment should be offered in an online, brief, structured group‐based format. The group format is aligned with the preferences of a majority (61%) of patients who face unsuccessful treatment,^47^ enabling them to share experiences in an empathic environment, learn from others' experiences and decrease feelings of loneliness. The online format is value by HCPs because it requires less resources from clinics (e.g., time, human resources) and by patients because it overcomes circumstantial barriers (e.g., travel costs) to access care. Indeed, the use of online video counselling seems the way forward to support patients after unsuccessful fertility treatment. Individual and group online video counselling are growing, especially since the COVID‐19 pandemic,^48^ and evaluative studies on its acceptability and effectiveness report promising results.^49^, ^50^, ^51^ However, results suggest that for a minority of patients, in‐person individual or group‐based formats may not be adequate. For these patients, online individual or self‐help interventions, for instance, www.myjourney.pt ^52^ may be preferable, as they ensure privacy, bypass possible stigma and offer more flexibility in access.

### 4.1 Strengths and limitations

This qualitative study used Bowen et al.'s^36^ theoretical framework to assess the acceptability and feasibility of psychosocial care for unsuccessful fertility treatment. The use of Framework Analysis enabled the preservation of participants' individual views and analysis of consensual and disparate views across different stakeholders, allowing for consideration of their specific needs. The qualitative process indicated that saturation was achieved, as the codes emerging in the final focus groups were anticipated by researchers and appeared to have no additional interpretive value.^37^ There were few Psy, which limits conclusions about their acceptability of support for unsuccessful treatment. Patients were mainly women, but men's participation in the present study was higher than usually observed in reproductive research^53^, ^54^ and male views were overall similar to female views. Patients were recruited at a single clinic and their views may not be representative of patients' experience at public fertility clinics.

## 5 CONCLUSION

Patients and HCPs perceive that clinics should improve their care provision for patients facing unsuccessful fertility treatment, but HCPs' misconceptions and concerns will need to be addressed to ensure signposting for preventive care while patients are still undergoing treatment. If interventions are to fit patients' and HCPs' needs and preferences, they should be brief and use online video counselling offered in group format. The *Beyond Fertility's* logic model was validated, which suggests that support at this stage should incorporate specific mechanisms of change: meaning‐making, acceptance and pursuit of new life goals, with additional emphasis on promoting social and partner connectedness. Future research should focus on developing and evaluating psychosocial care interventions tailored to this treatment stage.

## AUTHOR CONTRIBUTIONS

Sofia Gameiro, Bárbara Figueiredo and Mariana Sousa‐Leite contributed to the conception and design of the study. Sofia Gameiro and Mariana Sousa‐Leite contributed to the design of the study materials and executed the study. Salomé Reis, Mónica Fernandes and Mariana Sousa‐Leite contributed to the data collection. Sofia Gameiro, Bárbara Figueiredo, Raquel Costa and Mariana Sousa‐Leite contributed to the analysis and interpretation of the data. Sofia Gameiro and Mariana Sousa‐Leite wrote all versions of the report. Bárbara Figueiredo and Raquel Costa reviewed versions of the report, and all authors reviewed and approved the final version of the report.

## CONFLICT OF INTEREST

Dr. Gameiro reports consultancy fees from Ferring Pharmaceuticals A/S, speaker fees from Access Fertility, SONA‐Pharm LLC, Meridiano Congress International and Gedeon Richter, and grants from Merck Serono Ltd., an affiliate of Merck KGaA, Darmstadt, Germany.

## Supporting information

Supporting information.

## Data Availability

The data underlying this article will be shared on reasonable request to the corresponding author.
