# Supplementary material for: Feasibility and acceptability of psychosocial care for unsuccessful fertility treatment
Source: Health Expect. 2022 Sep 20;25(6):2902–13. doi: 10.1111/hex.13598 (PMC9700180; doi:10.1111/hex.13598)
Supplement: Supplementary file 1 — Supporting information. [file HEX-25--s001.docx]

Appendix 1

Focus Group Script (English Translation)

**Introduction**

Welcome to this discussion session. My name is Mariana, and I am a Psychologist and a PhD student at the School of Psychology of Cardiff University. We have with us today Dr Sofia Gameiro, from Cardiff University, one of the supervisors of this project. The discussion session, which we invited you to participate in, is integrated in a PhD scholarship, funded by the Foundation for Science and Technology, and was approved by the Ethics Committee of the São João Hospital Centre. We would like to thank your all for your participation in this session. Your contribution is highly relevant to the aims of this PhD project and so thank you for being here today, at this time, most likely after a long day of work.

We are here today to have the opportunity to get to know your point of view, based on your life experience (for patients) OR as healthcare professionals (HCPs) in the fertility field (for HCPs), about a psychosocial intervention programme for patients who face the experience of their last treatment cycle of In Vitro Fertilisation or Intracytoplasmic Sperm Injection (IVF/ICSI) being unsuccessful. One of the ways that we can understand the feasibility of implementing this programme and that we can improve it is by discussing it with the people who live and deal with the stress of this treatment on a daily basis (for patients) OR HCPs like you, who have contact with these patients on a daily basis and are most familiar with the way these patients cope with the experience of unsuccessful treatment (for HCPs). This discussion group is comprised by people who are preparing to or undergoing their last cycle of IVF/ICSI or who have undergone it for less than 6 months without success (for patients) OR by HCPs of different specialities, which will allow us to have a broader understanding of this experience (for HCPs). Discussing this programme with you allows us to have a deeper and more real understanding of how to address treatment. During this discussion I would like you to share all of your opinions, perceptions and suggestions regarding the development and implementation of this psychosocial intervention programme.

The World Health Organization currently considers infertility a public health issue. Research shows that a considerable percentage of patients end treatment without achieving a pregnancy. We know that IVF/ICSI fertility treatment is highly stressful and that ending it without achieving a pregnancy triggers an intense and prolonged grieving process, which is associated with short- and long-term mental-health problems and low well-being. Although several clinical guidelines stress the need of mental HCPs to provide structured and empirically validated psychosocial support to these patients after their treatment has not been successful, the truth is that there is no psychosocial intervention specifically developed to this end.

This was the reason why we are developing a psychosocial intervention based on scientific evidence, which aims to support patients who complete their last cycle of IVF/ICSI without achieving a pregnancy, promoting their positive adjustment. In particular, to reduce the symptoms of grief in the short and long term, to reduce the negative impact that fertility problems have on their life (at a personal, emotional and social level) and to promote the development of new perspectives of life, which should translate into better well-being, life satisfaction and mental-health.

This is a **brief programme**, of just seven sessions. The first two sessions are individual or for the couple, and the last five sessions are in group. The first session takes place while patients prepare to initiate their last IVF/ICSI cycle, the second session takes place one to two weeks after patients have received a negative result for this cycle and the following five are weekly and start four to six weeks after this result. It is very important to highlight that the **first session is** **directed to all patients** who undergo the last IVF/ICSI treatment cycle, **but** **all the following ones are ONLY directed to those who face the experience of this LAST cycle being unsuccessful.**

We have already some questions prepared to help guide this discussion in a more organised way, but please feel free to ask other questions or add other information that you consider important as we progress through this discussion.

It is very important that we keep everything you say, without losing any information. It would be impossible to write everything you say in full and in real time. For this reason, we asked if we could record this discussion. Please do not be concerned, this discussion is confidential, only the researchers of this project will hear the recording and it will be destroyed at the end of the project. We guarantee that you can stop and/or erase the recording at any time during the session. Does everyone agree that the discussion can be recorded?

We are here only to gather information, there are no right, or wrong answers and all opinions are equally welcome, even if they may be different or opposite, so we hope you feel comfortable to share with us what you really think.

We ask that everything you hear here today be confidential and not shared outside this discussion group. This discussion will last approximately 90 to 120 min., but in case you consider it necessary, we can take short breaks. We ask you not to interrupt others and for each one to speak clearly and in turn.

Does anyone have any questions before we start?

**Opening question (10 minutes)**

Self-introduction: name and a short curiosity they would like to share about themselves (for patients) OR profession + briefly explain their contact with these patients from the moment they prepare to initiate their last IVF/ICSI cycle (for HCPs).

**Specific questions:**

**Intervention demand**

Firstly, I would like to understand your position regarding the need to develop this psychosocial intervention programme…

1. Many patients face the experience of their last IVF/ICSI cycle being unsuccessful. How important do you consider it to be to support these patients in adjusting to this experience?
2. Prompts: reasons.

(Does anyone what to add anything else to this?)

**Intervention practicality**

Now, I would like to address a practical issue, quite important to the implementation of this programme…

1. What do you feel when you hear the name of this intervention: *Beyond Fertility*?
2. Prompts: do you consider the name appropriate; reasons; other friendly names.
3. Is it possible to identify the patients that are going to initiate their last IVF/ICSI cycle? **(only for HCPs)**

a. Prompts: in which moment is it possible to identify; how is it possible to identify - if they discuss with their patients about this being their last cycle, if so, when; what is the criteria - only related to the implicit criteria of the number of cycles reimbursed by the NHS.

(Does anyone what to add anything else to this?)

**HCPs’ needs (only for HCPs)**

Regarding the needs that you feel in your clinical practice…

1. In your day-to-day work life, what do you feel are your greatest needs or difficulties when interacting with these patients, and that you think this psychosocial intervention could help to solve or alleviate?
   1. Prompts: think, for example, in situations in which you would like to have done more, but for several reasons you could not, or that you had to ask for help from another HCP, in particular a mental HCP.

(Does anyone want to add anything else to this?)

**Patients’ needs before initiating their last treatment cycle - 1^st^ session**

Now, I would like us to talk a little bit about the first session, in which patients are preparing to start their LAST IVF/ICSI cycle...

1. From your experience, what do you feel that are/were your needs during the period you are/were preparing to initiate your last cycle of IVF/ICSI? (for patients) OR what do you feel are the needs of your patients during the period they are preparing to initiate their last cycle of IVF/ICSI and in your opinion this intervention should address?
2. Prompts: think about personal and social needs; in strained moments you might have had at the clinic with the fertility staff, your partner, family or friends (for patients) (for patients) OR with your patients (for HCPs); what themes may have caused this strain; what issues would you like to address during this stage and with whom (for patients) OR what issues your patients usually like to address with you during this stage (for HCPs); what are your most common complaints or difficulties during this stage (for patients) OR of your patients during this stage (for HCPs).
3. Preparing patients in advance for the possibility of their last treatment cycle being unsuccessful is one of the aims of the first session of this programme. I would like to hear your opinions on this aim.
4. Prompts: do you consider it important and acceptable - reasons; what would be the best time to initiate this discussion; what do you think would be the best way to introduce this discussion; what kind of information do you think should be provided; what resources or strategies could be used; how could we make this preparation easier for patients.

(Does anyone what to add anything else to this?)

**Patients’ needs after their last treatment cycle being unsuccessful - 2^nd^ to the 7^th^ session**

Now, I would like us to talk a little bit about the sessions after the last IVF/ICSI cycle being unsuccessful. Do not forget that the first session after failure is STILL individual or for the couple, but THE FOLLOWING FIVE ARE IN GROUP…

1. I would like to understand what contact you usually have with your patients after the failure of their last treatment cycle **(only for HCPs)**
   1. Prompts: immediately after and in the long term; frequency of contact; reason for contact; contact that they think these patients should have or would like them to have and with which HCPs.
2. From your experience, what do you feel are the greatest difficulties for patients after the last IVF/ICSI cycle being unsuccessful?
   1. Prompts: think about needs you may feel on a personal and social level and that you would like to overcome (for patients); in tense moments that you may have at the clinic, with the fertility staff, your partner, family or friends (for patients) OR tense consultations that you sometimes have with your patients (for HCPs); what themes may have caused this strain; what issues you think should be addressed during this stage and with whom (for patients) OR what issues your patients would normally like to address with you during this stage (for HCPs); what are the most common complaints or difficulties during this stage.
3. Literature shows us that it is very important to help patients to overcome the experience of their last IVF/ICSI treatment cycle being unsuccessful. I would like to hear your suggestions about the kind of support you feel would be useful to be provided in this programme and during this period (after the last IVF/ICSI cycle being unsuccessful).
4. Prompts: what are the most crucial aspects this intervention should address; what themes do you think should be addressed in this intervention; what strategies or resources could be used.
5. After the last treatment cycle being unsuccessful, our main therapeutic goals are: to promote acceptance towards the inability to have biological children, to reassess the efforts to achieve one’s parenting goals in a more positive way, to define new meaningful life goals, and to promote greater social support. What is your opinion on these goals? (presented by means of the *Beyond Fertility* logic model)
6. Prompts: reasons; do you consider some of these goals not to be so relevant; in your opinion is there any goal that you consider important and that has not been mentioned.
7. The last five sessions of the programme are in group. What is your opinion about this format?
8. Prompts: reasons; in your experience, do you think this is the ideal format to conduct the sessions after the last treatment cycle being unsuccessful; what alternative formats do you consider could be more beneficial.

(Does anyone want to add anything else to this?)

**Barriers and facilitators**

Finally, I would like us to talk a little bit about the implementation of this psychosocial intervention programme in public hospitals or private clinics...

1. If *Beyond Fertility* were introduced to you and made available during the period you are preparing to initiate your last IVF/ICSI cycle, would you consider engaging with the programme? (for patients) OR Would you be willing to promote this intervention programme in your hospital or clinic? (for HCPs)
2. Prompts: reasons; if not, which conditions would be required to be willing to engage/promote it.
3. If this psychosocial intervention was available, what could make it less attractive or hinder your engagement with it? (for patients) OR Which do you think would be the greatest barriers towards the implementation of this programme? (for HCPs)
4. Prompts: reasons; think for example about the experience you have had in the contact with the clinic or hospital (for patients) OR in terms of the clinic or hospital setting, the coordination between HCPs and the experience you have with the patients (for HCPs); how could we overcome these barriers.
5. How do you think we could facilitate your engagement with this psychosocial intervention programme throughout all the seven sessions? (for patients) OR How do you think we could facilitate the patient’s continued engagement with this psychosocial intervention programme (throughout the seven sessions)? (for HCPs)
6. Prompts: think, for example, in strategies or resources that have already been implemented and that have increased your motivation and commitment to other appointments (for patients) OR that have already been used with your patients and that have increased their motivation and commitment to the appointments (for HCPS).

**Closing question**

Is there any further information about what we have discussed today that you would like to share before we finish?

## Appendix 2

**Supplementary Table 1**

Framework matrix: one row per theme and category, and one column per group (patients; psychologists; fertility specialists)

|  | **Patients (*n* = 13)** | **Psychologists (*n* = 3)** | **Fertility specialists (*n* = 6)** |
| --- | --- | --- | --- |
| **THEME: High demand for psychosocial care across the whole treatment pathway** | | | |
| **Category: Fertility treatment is highly challenging** |  |  |  |
| Fertility treatment is highly challenging for patients, and its failure triggers an intense grief process | Patients reported a high desire to reach parenthood and, three patients added that both members of the couple had the same high desire.  The fertility treatment process is physically and psychologically demanding (and financially expensive in the private sector), and its failure triggers intense grief emotions and thoughts.  “*it has not been an easy process at all, quite the contrary, each treatment is increasingly difficult, psychologically it has been a drastic shock. I’ll even, I’ll just give a small example, in the last negative treatment I had, I felt so bad, so bad, that when I was going to the subway, I tripped on the escalators and fell and almost broke my knee. That’s it, (...) but I think in these situations [unsuccessful cycle attempts] we [patients] are all in it together, aren’t we!?*” (Pa1)  Each cycle is perceived as more challenging - most patients need to cope with previous unsuccessful cycles and subsequent waiting lists. The waiting list period and the two waiting weeks for the beta hCG test result tend to be most stressful.  “*That time [waiting list] was a torture because I didn’t know what to do, I didn’t know if, if I would receive treatment because they didn’t guarantee either, right!? So, I got a little lost.*” (Pa1)  Patients (primarily women) identified negative emotions and feelings experienced during treatment (which tend to intensify by approaching the last cycle and/or age limit to undergo treatment) and after its failure - anxiety, anguish, sadness, denial, confusion, guilt (for not being able to conceive), envy(friends when they reach parenthood), frustration, unconformity and shame, loneliness, despair, lack of self-compassion, lack of control, revolt, frustration, and relief after unsuccessful treatment due to the end of the tension. Women also felt that the process impacted their self-perception, in particular, identity conflicts, lower self-esteem, four women related with the self-perception of being spoiled or expired, and three women perceived a negative body change. One man reported feeling unsupported and invisible in fertility care.  “*this tension [during treatment] is so, it’s so strong, it’s so much more intense than what we can explain.*” (Pa2)  “*I don’t know. I can tell you that I don’t know what’s going on with me. There are times I think I’m normal, and there are times I’m not normal and now, and, and I don’t even know if it’s because of that [unsuccessful treatment]. But I feel different (...) for instance, I was a woman who has never needed psychological support for anything and now [after unsuccessful treatment] I find myself in a riot.*” (Pa2)  Five patients have also indicated that this process is characterised by several fears, for instance about: having a negative beta hCG test, the future of their relationship as a couple in the eventuality of this outcome, inability to give a sibling to their child, and how pregnancy evolve in case of a positive beta hCG test.  “*wow, completely, what is this [unsuccessful treatment] going to do to us, right!? to us as a couple. What is all this project going to do (Pa2: of course).*” (Pa3)  Despite almost all patients agreeing that women have the additional burden of dealing with the physical and hormonal component of treatment, four patients added that women were more willing to continue undergoing treatment than their counterparts.  “*It will only be my last [cycle] if they [clinic] tell me: your body can’t take it anymore.*” (Pa4)  Due to this emotional burden, one couple has asked to postpone their treatment cycle. | One psychologist referred that during treatment, reaching parenthood is, for most patients, their single life goal. The period after unsuccessful treatment is an intense process of grief (with one psychologist stressing that for most patients, this grief process starts before the end of treatment, as patients progress throughout it and face unsuccessful outcomes, and sometimes is about fertility and not parenthood) - patients tend to experience frustration, anger, hopelessness, denial, and guilt for not being able to conceive. Two psychologists referred that patients wanted to leave the clinic as soon as possible after unsuccessful treatment due to the emotional burden treatment entails.  “*the emotional storm they [patients] are in, because several times the emotional distress is brutal at that time [after unsuccessful treatment], and there is often a void, there is a big void about the future.*” (Psy1)  “*Even when there is no psychopathology, it’s a grieving process they [patients] will need to undergo, isn’t it!? And it’s the end of a process, a process that sometimes was very long.*” (Psy2) | One GYN/OBS referred that during treatment, reaching parenthood is, for most patients, their single life goal. Three fertility specialists agreed that patients who undergo multiple unsuccessful cycles adjust to the last cycle (and its failure) in a different way than those who only undergo one cycle, with one GYN/OBS referring that these patients start their grief process before the end of treatment as they progress throughout it and face unsuccessful outcomes. However, one GYN/OBS highlighted that the last cycle is the most challenging one and in which patients have more significant emotional difficulties and needs. Over treatment and after its failure, patients experience intense negative emotions - sadness, frustration, anger, lack of control, and revolt, and one GYN/OBS sometimes perceived some aggressiveness towards them. Two fertility specialists referred that most patients end treatment with many questions about their fertility problems and unsuccessful outcomes and explain them. Two fertility specialists referred that some patients ask to postpone treatment cycles or discontinue treatment due to the emotional burden it entails or having ended their couple relationship.  “*approaching the last treatment [cycle] or before the last treatment [cycle], which may or may not be unsuccessful, in the meantime it has already been, or may have already been 3 or 4 years, right!? And there is an accumulated burden for the couple, accumulated suffering. So, I think when the couple approaches the last treatment [cycle], indeed, it is probably the time of greatest distress because time goes by*.”(Fs1) |
| Undergoing fertility treatment leads to significant mental-health problems for a minority of patients | One woman referred that her anxiety symptoms become worse and more frequent over treatment. Another woman referred she has started to have panic attacks, and another had begun to take anxiolytics and antidepressants.  “*I think during that waiting time [waiting list], I psychologically got fed up with crying. I got fed up with having anxiety crises, something that I have never had, I never had. At this moment, anxiety crises and panic attacks are part of my daily life and even, and until recently, I didn’t know what it was*.” (Pa1) | One psychologist reported that the hospital has already received suicidal attempts after unsuccessful treatment. | One GYN/OBS referred that some patients develop psychiatric disorders during treatment or exacerbate their symptomatology. One nurse stressed that the hospital has already received suicidal attempts after unsuccessful treatment.  “*we’ve also had some attempted suicides post unsuccessful treatments (…) So things go up to this point.*” (Fs2) |
| Relational interactions are also challenging to manage for patients | Patients agreed that treatment is challenging for the couple, with one patent referring it did not negatively impact their couple relationship and two patients referring it did. Patients tended to restrict the number of people aware of their fertility path, particularly in the last treatment cycle. All patients agreed that managing others’ insensitive comments and expectations towards parenthood and fertility treatment (from family, friends, and work colleagues) is challenging and distressful, feeling misunderstood by them. Most women also added the high visibility they experience at work due to their frequent absences due to treatment and consequent need to justify. Four patients also claimed stigmas related to fertility problems and treatment and stressed raising awareness about fertility problems.  “*maybe this was exactly what I needed, to talk with different people, people who didn’t have, didn’t accompany me and, and some of them who went through and are going through the same experience as me. That’s what I needed, and I think I feel a lot lighter.*” (Pa2)  “*Because sometimes our family circle or our friends aren’t going through the same situation, and sometimes you want to talk, and they don’t understand (Pa7: it’s true). They downplay a lot what you’re feeling, and, at that moment, you feel even angrier (Pa7: it’s true).*” (Pa5)  “*and then returning to work is very difficult, is terrible [crying], chatting and facing people again is also complicated.*” (Pa3) | Two psychologists referred that most patients tend to restrict the number of people aware of their fertility path (some do not tell anyone), explaining that patients do not feel understood by others (family and friends) and that it is difficult to manage their insensitive comments. One psychologist referred that there are still stigmas associated with fertility problems and treatment. One psychologist also highlighted that many patients have several work absences to undergo treatment.  “*many of them [patients] have this thing, which is: even when they tell their family and friends, they say: -Oh, yeah, it’s going to be this time, it’s going to be all right, you’ll see. And then they say: -yes, you know psychologist, but they don’t understand anything of what we’re feeling, do they!? I mean, they want to support us, they care a lot about us, but we can’t explain to them what we are feeling because only those who go through this truly understand.*” (Psy2) | Two fertility specialists referred that most patients are unaware of the high prevalence of fertility problems until they enter the waiting room. Two specialists referred that the couple relationships is often negatively impacted by treatment, with some couples having communicational issues and/or disagreement about the treatment process. Two specialists also perceived some stigma associated with undergoing fertility treatment, particularly those patients who undergo treatment with donated gametes (i.e., afraid of being recognised in the clinic). One GYN/OBS highlighted that many patients have several work absences to undergo treatment.  “*when they [patients] arrive to start the first treatment (…), they say: we didn’t know there were so many people going through the same situation as us. We thought we were the only ones, that we were the only ones, but then we get here, and the room is full of people*.” (Fs3) |
| Patients’ adjustment to unsuccessful treatment is associated with individual and treatment-related factors: patients’ individual characteristics, quality of their couple relationship, number of previous unsuccessful cycles, and receive in-depth scientific medical explanations for their fertility problems, treatment choices, and unsuccessful outcomes. | Patients perceived that their adjustment to an unsuccessful cycle result depends on their individual characteristics and resources, quality of and communication within their couple relationship, expectations towards the outcome, and in-depth medical explanations for their fertility problems, treatment choices, and unsuccessful outcomes.  “*this is very important in, in the grieving process, at least we feel it is, why did this happen?*” (Pa6)  “*it was something [the communication of a negative Beta HCG result], it was something that we, that we felt and that was very tough, in particular in the first one [cycle] (Pa3: yes, it was), and it’s because of all those expectations we bring, isn’t it!?*” (Pa6) | Patients’ adjustment to unsuccessful treatment depends on their individual characteristics and resources, emotional stability, the quality of and communication within their couple relationship, and the number of previous unsuccessful cycles.  “*The other aspect sometimes is having the perception that the couple is a vulnerable, fragile couple, who possibly have few resources. There are several risk factors from an emotional point of view, and sometimes a prior work before treatment would be beneficial for those couples*.” (Psy1) | Patients’ adjustment process to unsuccessful treatment cycles (including the necessary time to initiate another cycle) depends on the patients’ individual characteristics and resources, emotional stability, the communication within their couple relationship, the number of previous unsuccessful cycles and treatment prognosis, expectations towards the outcome, quality of the HCP-patient relationship, and having in-depth scientific medical explanations for their fertility problems and unsuccessful treatment outcomes.  “*If it [fertility treatment] doesn’t go well, the fall, and of course if this is a first and last cycle even worse because they know it’s the only possibility they have, from this point of view. Here the fall, and from a psychological point of view, can be dramatic*.” (Fs4) |
| Patients tend to find their own coping strategies to manage treatment as time goes by. The first weeks after unsuccessful treatment are the most challenging ones for patients, but HCPs claimed that patients tend to adjust over a 1- to 2-years period. | Four patients referred that women and men have different coping strategies to deal with the treatment burden and its failure. Most patients referred that they started treatment with unrealistic high expectations but tended to downgrade them over time as they experienced unsuccessful cycles. Three patients referred feel more at ease with their fertility path over time, and almost all indicated they had found beneficial coping strategies to deal with it, as restricting the number of people who know about their treatment path, seeking pleasant activities and career successes, discussing their future as a couple in case of an unsuccessful cycle, and taking some time to focus on themselves. Three patients referred that it was beneficial to have the opportunity to share their experiences with other fertility patients, and four patients have started to consider different paths to achieve parenthood (adoption or donated gametes/embryos). Three patients referred to positive consequences as a result of their treatment path: have learned with the process and/or grow as individuals and couples.  “*this was a conversation and a very big fear (…) which was indeed this conversation we both had, very serious, what if, if we never make it, huh!? What’s going to happen to us [as a couple], you know? And I think it’s very important.*” (Pa3)  “*it is in the other slices that we find the strength to continue fighting after all these years: in family, work, achievement, contribution to society, all slices of the wheel of life, as they say, all, all these slices because that’s what complements us.*” (Pa7) | Patients start treatment with unrealistic high expectations but tend to downgrade them over time as they experience unsuccessful cycles. During this process, patients find beneficial coping strategies to manage the process - restricting the number of people who know about their treatment path or only sharing with know-other fertility patients, considering other paths beyond biological parenthood: childfree lifestyle, adoption, or donates gametes/embryos, and seeking online discussing groups. One psychologist referred the first weeks after unsuccessful treatment are the most challenging ones for patients, but they tend to adjust over a one- to two-year period.  “*but in my experience, whenever I talk to the couples in a sense they could seek, I mean, other couples with whom they could share, they do that, but through the technologies, the Internet, they look for discussion groups*.” (Psy1)  “*As a matter of fact, we notice this, couples who have someone they know who has gone through the same [treatment process], that couple turns out to be a great support and someone with whom they share a lot of this path, isn’t it!?*” (Psy2) | Patients start treatment with unrealistic high expectations but tend to downgrade them over time as they experience unsuccessful cycles. During this process, patients find their own coping strategies to manage the process - restricting the number of people who know about their treatment path and/or considering other paths in advance: childfree lifestyle, adoption, or donates gametes/embryos. Two specialists referred that after unsuccessful treatment, the first weeks are the most challenging ones for patients, but they tend to adjust over a one- to two-year period.  “*indeed after one year or they have already moved on, or they don’t even want to hear about this anymore, because they’ve already, already moved on into a different direction*.” (Fs1) |
| **Category: Covid-19 pandemic made treatment even more challenging** | The covid-19 pandemic was an extra anxiety factor for patients. Five patients referred increased waiting for periods and uncertainty about access and time of future cycles.  “*and that’s it, one more thing to make me anxious: the covid-19.*” (Pa8)  “*I was supposed to do my last cycle in the month of May/June of 2020, but (...) due to covid, it was postponed for a month, two, three (...) until this month [January].*” (Pa1) | One psychologist referred that due to the covid-19 pandemic, all psychosocial groups in other health fields were on stand-by.  “*In our hospital, for example, we had groups functioning, as I was previously saying with the pregnant women in obstetrics, and the groups are suspended for now*.” (Psy2) | Due to the covid-19 pandemic, the fertility clinic reduced the patients’ physical presence to compulsory procedures, and some clinics started to give the pregnancy test result by phone. One GYN/OBS highlighted that some of these adopted new dynamics might be worth keeping in the future.  “*at this stage due to covid, this is not happening, right!? We are the ones who call to give the results, or the couple sends us the result first, and then we confirm the result, so to speak.*” (Fs3) |
| **Category: High demand for support at all stages of treatment, but particularly after it being unsuccessful and in a group format** | All patients stressed there is a high demand for psychosocial support. This support should be offered to all patients (both members of the couple) during all treatment process stages, with five patients highlighting it is particularly important at later stages of treatment.  “*my biggest trauma was when they gave me the negative result (…), and I think from that moment on I should have received support.*” (Pa1)  “*I received a big no, and I think that regardless how much strong I think I am, if I had psychological support, at least in the end…*” (Pa2)  “*I think the couple doesn’t need to receive psychological support only when they know the negative test (…) there is already pressure before treatment, not just after it. And that [pressure] accumulates, and when you have a negative test [result], then the world falls on you.*” (Pa5)  “*I think it’s also important, as you said, to provide support for men, which is a little forgotten very, very often (Pa2: it’s true).*” (Pa9)  Patients emphasised the importance of receiving support in a group format. Two patients stressed the importance of receiving it from mental-health professional experts in fertility. Several benefits of receiving psychosocial support in a group were identified: it would decrease patients’ feelings of loneliness and be a space where patients could share their experiences with other fertility patients, listen to their views and experiences and learn from them.  “*sharing with other women, trying to understand other couples, trying to understand if the fears are the same, if they aren’t, what they did what they didn’t do, some strategies that can help us, I think it would be important.*” (Pa3)  “*Yes, I think sharing is important as well, and realising we are not alone, we are not the only ones going through the same situation. Yes, I fully agree*.” (Pa13)  “*I think it’s positive to learn about others’ perspectives and experiences. For instance, I would never have imagined your story could be possible (…), and I think this is very important, it opens our minds, opens our horizons, doesn’t it!? And I also think it’s good for us to have… to know there are more people with problems like ours, isn’t it!?*” (Pa8) | Psychologists stressed that psychosocial support is essential for fertility patients at all stages of treatment, in particular after unsuccessful treatment. All agreed that some patients are in more need of support, particularly those who have less emotional resources, are more disorganised as a couple, and/or resistant to receive it. Psychologists emphasised the importance of receiving support in a group format, stressing several benefits: decreasing patients’ feelings of loneliness and an opportunity for them to share their experiences with other fertility patients who understand their experience. One psychologist stated that although their psychology department desire to organise these groups, no fertility group support options are available.  “*Indeed, it’s something that we at psychology in the clinic have been thinking about for many years [creating psychosocial support groups], if it makes sense if it doesn’t, because we have support groups in other areas (…) and I think they [these other groups] work very well.*” (Psy2)  “*And in this regard, being able to be with couples who are going through the same, in particular during this stage of failure and grief, I think it might have, it has a powerful therapeutic aspect, hasn’t it!?*” (Psy2) | Fertility specialists stressed that psychosocial support is essential for fertility patients at all stages of treatment, in particular after unsuccessful treatment. Five specialists referred that some patients are in more need of support, as those who are more disorganised as a couple, socially isolated, undergoing treatment with donated gametes/embryos, and/or show higher levels of psychological distress. Fertility specialists emphasised the importance of receiving support in a group format. Three specialists highlighted its benefits: decreasing patients’ feelings of loneliness and an opportunity for them to share their experiences with other fertility patients who understand their experience.  “*I think it should be mandatory to have psychology integrated in the fertility department (…) because no matter how well they [patients] are supported at a scientific and technical level if they are not at a personal and emotional level, there will be a gap there.*” (Fs5)  “*most importantly, when we finish this process [fertility treatment] and things do not go well, and we no longer have a connection [with patients], I think psychological care is essential*.” (Fs5)  “*I think group intervention could have the benefit of enabling couples to see that after all, they are not the only ones and that indeed there are many other couples who, who have gone through the same as them and will also have to find other strategies*.” (Fs1) |
| **Category: Opposite views expressed between patients and HCPs about accessibility of support** | Only one couple claimed they were referred for support in the fertility clinic by the nurse after the female partner discussed the emotional burden the couple was going through. This couple referred they were dissatisfied with the support provided and discontinued it after one session: they felt the support was only offered because it was mandatory. They feel misunderstood, as the support was not adjusted to their needs - composed solely of general information provision and behaviours imposition. All the other participants referred that they were not offered psychosocial support, not knowing which support they have available for them. One patient and the couple mentioned above who were receiving or received support self-referred outside the clinic. These patients indicated that it helps them manage their couple relationship, accept their negative emotions and feelings, and cope with their fertility path.  “*I’ve been accompanied by a psychologist for a year and a half, by my self-recreation, because no one has ever signposted me, neither in private [clinics] nor here, and of course it’s helping me a lot*.” (Pa4)  “*I think it would be important the [fertility] department have psychology integrated. I do not know, if they have it, they didn’t tell me anything (…) as Pa4 said a while ago [“no one has ever signposted me, neither in private [clinics] nor here”], no one asked me anything, no one asked me: do you need it?*” (Pa8)  “*to whom can I turn to? What is the psychological support that I have available for me?*” (Pa1)  “*so, I thought there was [support received in the clinic] imposition of behaviours, wasn’t it!? it has to be done in this way, it has to be done in this way, and maybe before it has to be done in this way, it has to be understood why we are doing it in the other way, isn’t it!?*” (Pa7) | All psychologists referred that support is available to all fertility patients. Two psychologists indicated that their fertility clinics integrate compulsory sessions: in one clinic, all fertility patients who initiate fertility medical appointments and/or treatment with donated gametes/embryos are directed to a mandatory initial psychosocial evaluation session, and in another clinic, this process happens with all patients referred to initiate their first IVF/ICSI cycle and/or treatment with donated gametes/embryos. These two psychologists stressed that the option to turn to psychosocial support at any time is offered to all patients in these appointments, and continued support is offered to those who show significant emotional distress. At later stages of IVF/ICSI treatment, only patients who present significant emotional distress are referred to support by fertility specialists. During the last treatment cycle and after its failure, all psychologists claimed they have almost no contact with these patients.  During support, psychologists focus on conveying realistic expectations towards the treatment outcome, facilitating patients’ grief and adjustment process and/or facilitating couple mutual support and communication.  “*we have some couples that will initiate the last cycle or after the last cycle that are accompanied in the psychology appointment. But, roughly, I think it is something around 10%, 8%, something like that.*” (Psy2)  “*Then, our intervention when the couples are preparing to initiate IVF/ICSI treatments, then usually the couples who come to us are always those who present significant emotional distress, which is somehow interfering with the treatments being carried out by the medical team*.” (Psy1) | All fertility specialists agreed that psychosocial support is always available - all patients could ask for it at any time - although they only refer to support patients at more risk for maladjustment, showing high levels of emotional distress and/or couple communication conflicts. Notwithstanding, one GYN/OBS directed that in their clinic, all patients referred to initiate their first IVF/ICSI cycle or treatment with donated gametes/embryos are referred to a compulsory psychosocial evaluation session.  “*when the situation is clearly serious and requires the intervention of a specialist in the area, then yes, only a psychologist will be able to truly support the patient. Indeed they should always be there, but when it is not possible, perhaps we try to do a little of what could be an essential help in this part*.” (Fs5)  “*when we notice there may be a need for psychological support, so of course, in that case, we take advantage of that, and this has already happened - referring to the psychologist so there can be a follow-up*.” (Fs4) |
| **Theme: High acceptability of preventive and early psychosocial care** | | | |
| **Category: Ambivalence in preparing patients in advance for the possibility of unsuccessful treatment** | All patients referred they feel misinformed about the fertility process - their fertility doctor does not respond to their informational needs, provides lack of in-depth scientific explanations for their fertility problems and failed cycles, and patients feel they need to run after their doctor to receive information - which impacts patients’ emotional adjustment during and after treatment. Indeed, three patients perceived their diagnosis was not in-depth studied and that fertility specialists use a trial-error method for each cycle.  “*it’s a confidential service, very confidential (…) we got there and what we are going to do now and, and what we are going to do next. Some doubts remain between, between appointments, between, between [treatment] stages*.” (Pa10)  “*And, being there, waiting without any information, is as if they were killing us a little, more and more*.” (Pa11)  “*no one can give us an answer about why this happens, why this does not happen. This is very important in, in the grieving process, at least we feel it is*.” (Pa6)  Four patients also perceived that the fertility medical doctor conveys unrealistic high expectations of the treatment outcome, and five patients highlighted and agreed that the fertility department setting also focuses on the positive side of treatment.  “*the doctor when, when she came to me at the end, saying: -yeah, it was very good, we got 16 oocytes, wonderful. We were left with an expectation, huh, huge, huge (…), and sometimes it doesn’t mean that, and I think we need someone who would also psychologically support us (…) so that we could be prepared for this as well, right!?*” (Pa1)  “*we were, as the colleague also said earlier, an easy case, from that easy case we became a difficult case, for 9 years.*” (Pa11)  “*we got there [clinic], and at first we go full of dreams, and everything will be fine because we get there, and we see lots of pictures of babies that were born there, and it’s all very beautiful and wonderful, and then we receive a NO.*” (Pa3)  Six patients agreed that the way the fertility team communicates the negative beta hCG test result is insensitive and impacts patients’ overall adjustment. Five patients stressed that training fertility staff on patient-doctor interpersonal relationships is highly needed.  “*My greatest difficulty in my last negative treatment was the day I knew the result had been negative. The way they told me: look, your treatment was negative, now you go home, wait, and in a year we call you again. And that’s it*.” (Pa1)  “*I think it would be really important to take this opinion of, of sensitising to the communication of the bad news, right!? Because sometimes this first, this first impacts, sometimes dictates everything, doesn’t it!? (…) I believe this communication [of the negative beta hCG test result] in the last treatment [cycle] is terrible, and I believe it is very important for the professional who gives it to know how to covey it, in the best way, right!?*” (Pa3)  Before initiating the last fertility treatment cycle, most patients would like to be prepared for the possibility of an unsuccessful treatment (with five patients adding it should be carried at later stages of treatment - after the second cycle being unsuccessful) in a realistic and balanced way, so not to impact their engagement with treatment. This support should prepare patients for what might happen during treatment, help them to manage their fears, anxiety, and expectations regarding its outcome and consider their future as a couple in case of unsuccessful treatment, should reassure patients that psychosocial support is always available for them (how and where they can have access to it), and be tailored to patients’ individual characteristics and preferences (non-compulsory format). Three patients also referred the need to prepare patients for the pregnancy period in case of a positive beta hCG test result (i.e., common emotional reactions and adverse outcomes).  “*a very big fear (..) what if, if we never make it, huh!? What’s going to happen to us [as a couple], you know!? And I think it’s very important for the couple to work on it as a couple. I am sorry, sorry I’m already, I’m falling apart (Pa2: me too) [emotionally activated]” (…) maybe it is important for you and your partner to talk about it, you have this fear, but he has it as well, isn’t he?” (Pa3) -“of course.”* (Pa2)  “*address these fears but also expectations, isn’t it!? What Pa2 was saying just now, to give some optimism, but a balanced optimism, because we can’t go, right!? as I went to my first treatment, thinking: Wow, now this is going to be, and then a bomb drops like that, and we’re completely barefoot and floorless*.” (Pa3)  “*I think we need to know where to go if our treatment is negative, right!? For instance, my treatment is negative, I can’t, I don’t know, I can’t do more treatments, I might even not come, not have, not, not come to have children, who can I contact? What psychological support do I have available for me?*” (Pa1)  “*I agree with Pa5 and P10, we really need a lot of support to manage our expectations.*” (Pa1)  Only three patients were unwilling to be prepared for this possibility, as they think the information they had was enough, it is a moment for patients to be focused on the positives, it could be distressful for them, and/or there is no point in being prepared for something that might not happen.  “*I don’t know if it would not be stressful, at that stage, before treatment, to be contemplating this possibility when the door is not yet closed”* (Pa6). *-“No, I don’t think so either. I don’t think so, it’s not time, it’s time for us to have all our strength up, with our good mood, our optimism, our hope.*” (Pa2) | Two psychologists indicated that patients are informed about the treatment success rates and prepared for the possibility of undergoing multiple cycles in the initial compulsory psychosocial session, but referred that patients tend to do not believe the information they convey at this initial stage.  “*I have, indeed, been confronted when I am with those couples who seek the psychology appointment again, they end up saying: -look, a lot of what you said in the first appointment we heard, but it didn’t make much sense to us. We, we remembered everything you said when we were actually confronted with the first failure, with the second failure, with the third failure*.” (Psy1)  All psychologists referred that it is important to convey realistic and balanced expectations towards the treatment outcome, and two psychologists claimed that it is important to prepare patients for the possibility of its failure - prepare patients for what might happen during treatment, give them strategies to cope when and if failure actually happens, work on their relationship as a couple (communication and support) and considering their future together, considering simultaneous alternative goals, and reassure patients that psychosocial support is always available for them. This preparation should be adjusted to the patient’s individual emotional and cognitive resources, and one psychologist stressed the importance of not focusing solely on the negative aspects. One psychologist also referred that it is important to address fertility problems as a health problem to decrease patients’ feelings of guilt and responsibility.  “*But I think we have to adapt the information to the couple we have ahead, right!? Listen to them first and also try to understand how they integrate this information*.” (Psy2)  “*I was thinking about maybe asking couples to give a speech about how they see themselves in two years or three years. The scenarios the couples see for themselves as a couple*.” (Psy1)  Only one psychologist was unwilling to prepare patients for the possibility of unsuccessful treatment, advocating that it might increase patients’ emotional distress - at this stage, it is important to acknowledge and normalise patients’ emotions.  “*So, therefore, they don’t have to be happy, because it hasn’t happened yet and they won’t pretend something they don’t feel, but they can’t be crying as if it had already happened, because that’s what emotionally destabilises them more at the moment. So it’s important to recognise the emotions and the anguish and the anticipatory anxiety of failure and accept that as normal, as legitimate*.” (Psy3) | During treatment, HCPs perceive they clarify patients about the treatment process.  In practice, mixed approaches to manage patients’ expectations towards the treatment outcome were reported. One GYN/OBS indicated that they tend to convey a sense of hope to their patients and try to focus on the positive outcomes, another tends to focus on the exact success rates, and one nurse reported they tend to convey low expectations to decrease patients’ high expectations. Two fertility specialists stressed that patients tend not to believe in the low treatment success rates they communicate at the initial stages of treatment.  “*I always try to show them the worst-case scenario when they come out very positive [laughs]. I don’t know if it’s the best or worst strategy, but that’s what I try to show. Most of the time: it will go wrong, 10% here, 20% there, 30%, 80%, 90% is negative.*” (Fs2)  “*I do the opposite (…) I think if people come for a treatment and assume right away that they’re going to have a failure, yes, of course, a treatment has between 30 and 40% of success, so it has 60% of failure, but 40% is not that low, is it!? And I think we can’t be, I’m an optimistic person, by nature, so I think, I try to convey that optimism to patients.*” (Fs1)  All fertility specialists referred that it is important to convey realistic and balanced expectations towards the treatment outcome (with two fertility specialists highlighting the importance of communicating the exact success rates), and prepare patients for the possibility of its failure - give patients strategies to cope when and if failure actually happens, help them to find alternative fulfilment lifegoals beyond parenthood and recognise and focus on the positive things patients have in life. One GYN/OBS also highlighted the importance of addressing the social-related issues about having a child with donated gametes/embryos. Notwithstanding, fertility specialists highlighted the importance of maintaining patients’ hope towards the treatment outcome.  “*Therefore, the more realistic expectations we give the couple, the easier it will be to deal with the couple, in terms of the treatment results, and this is visible. In my personal experience, if we prepare them well and manage their expectations well, this couple will manage to deal with it even if they have a negative result. And this, and this role for us is crucial*.” (Fs4)  “*although we can’t in an appointment, assume that this is going to be a failure, right!? We have to assume that we are doing treatment, and therefore we have expectations that things can go ahead, but we also have to open a window for failure. And before they [patients] are confronted with failure, they must realise there are other things beyond that [parenthood]. So, then they don’t focus only on that point, which isn’t the only goal in their lives*.” (Fs1) |
| **Category: Different perception between patients and HCPs about patients’ willingness for support after unsuccessful treatment** | All patients were willing to receive psychosocial support at any stage of treatment. Patients agreed support should be offered immediately to two weeks after unsuccessful treatment, with two patients stressing it should not be provided too late after it and three indicating the time needed might depend on the patient’s individual characteristics.  “*like any disease that manifests by itself, any symptom that manifests by itself, the more, the earlier it is approached by professionals, the better the perspective of resolution of the situation would be.*” (Pa6)  “*I think it’s important, each one will feel the need to, to talk again and approach things in a different way, but it’s also important to give it time to acknowledge things, isn’t it!?*” (Pa7) | Two psychologists claimed that most patients are not willing to receive psychosocial support after unsuccessful treatment. Patients want to close the process and leave the clinic as soon as possible, as they feel overwhelmed, hopeless, and sometimes angry due to the intense emotional burden that treatment entails. In some cases, patients call the service later and ask for support (primarily women).  “*What happens, from my experience, is that most times patients are not willing to [psychosocial support after unsuccessful treatment], they are very frustrated, very angry, very discouraged*.” (Psy1)  “*indeed, as the colleague said, when couples are not going to undergo any more treatment, usually they quit, they abandon us so to speak, so even if support would be offered, they are in a stage of some, some, saturation, and also disappointment and in the most immediate moment do not request this support*.” (Psy3)  Overall, psychologists have more contact with the female partner, as the male partner is less willing to receive psychosocial support.  “*we have two types of couples in the appointments, we have the couple who always come together and which are a minority, they are really a minority and I, and I remember almost all of them, so few they are (…) the tendency is for women to come alone*.” (Psy2)  One psychologist referred that psychosocial support should not be offered immediately after unsuccessful treatment, as patients might not be emotionally prepared, and suggested a period of around one to two months after it. Notwithstanding, agreed that patients might feel unsupported if provided much later (up to 1 year).  “*month and a half, I think so (…) otherwise they would feel helpless.*” (Psy1) | One GYN/OBS referred that after unsuccessful treatment, when the GYN/OBS offers the possibility of receiving psychosocial support, patients tend to not accept it due to the intense emotional burden they are going through. In some cases, patients call the service later and ask for support (primarily women).  “*in the last appointment in which the test result is given, we sometimes see that people are a little disturbed, and we even ask: -look, you can book an [psychosocial] appointment outside. And sometimes we see the couple don’t want to (...) But then sometimes, if we leave that option open, sometimes they call back later. Or so sometimes there is no acceptance from the couple, but then the woman ends up calling afterward*.” (Fs1)  Overall, fertility doctors have more contact with the female partner, as the male partner is less willing to go to the appointments.  “*As the medical doctor (Fs4**)has already said, for some couples it’s already difficult to have them [both members of the couple] in the [medical] appointment, isn’t it!?*” (Fs6)  Fertility specialists agreed that psychosocial support should not be offered immediately after unsuccessful treatment, as patients might not be emotionally prepared, and suggested a period of around one to two months after it. Notwithstanding, two fertility specialists also agreed that patients might feel unsupported if provided much later (up to 1 year).  “*a one- or two-year period, I am sorry, it seems too long (…) they [patients] might no longer need the psychological intervention*.” (Fs1) |
| **Category: Promoting acceptance and pursuit of positive and new life goals after unsuccessful treatment** | After unsuccessful treatment, patients indicated several needs they believe psychosocial support should address: helping patients to identify, express, and accept their negative emotions and feelings (for instance, frustration and women’s lack of self-esteem), and to manage social pressure and others’ expectations and comments (from family, friends, work colleagues) towards parenthood and fertility treatment (needs experienced across the whole treatment path), helping patients to accept their inability to have biological children, acknowledge and value the positive things they have in life beyond parenthood, work on their future as a couple and/or find other alternative fulfilment projects and/or paths (e.g., adoption, childfree lifestyle) beyond biological parenthood.  One man also indicated his need for help to support their female partner in their grief process.  “*I don’t know what I’m feeling (…) [a support to] help us to interpret ourselves, about what we are feeling, on, on how to react (…) I think it would be very, very important*.” (Pa2)  “*We need to know, and we need to know how to deal with each other as a couple after this, because it’s been a lifetime thinking that this will happen sooner or later, and suddenly there’s that, no, it’s just the two of us*.” (Pa2)  “*I also point frustration management first, right!? It’s a failed project, even though they tell us [it’s not]. And I think psychological support could help us find other projects, right!? Which none will replace [biological parenthood], but which could help, I don’t know, try to help us find another life project, which does not involve motherhood. And, and so towards the bottom of the pyramid, but I also think it’s important to help us to manage social pressure*.” (Pa4) | All psychologists indicated that support after unsuccessful treatment should facilitate patients’ grieving process: helping patients to identify alternative fulfilment lifegoals beyond biological parenthood, recognise and focus on the positive things they have in life and that have resulted from treatment, accept their inability to have biological children and/or manage difficult social interactions related with parenthood.  “*working on the desires, which desires are possible, so that the couple can see, from the point of view, which desires that woman can have about her life, which [desires] she can seek, or develop or discover, whether the woman or the man and in particular what makes sense for the couple*.” (Psy1)  “*When you [patients] arrived here, you had a life, where is it? So, there were many things, now you think this is the most important thing in your life and that without it nothing will work, but so far there have been many things that were once more important in your life and that cannot or should not be lost because of lack of investment*.” (Psy3)  “*teaching them to manage the issue of being with other couples, friends, and family, who have children and they don’t, and no longer have the hope indeed, so live with the idea that after all, they won’t have*.” (Psy3)  One psychologist referred that in their clinic, there is an additional short-term medical appointment after unsuccessful treatment for patients to ask questions and clarify their process, which is perceive as beneficial to facilitate patients’ grieving process.  “*as the doctor said, that last contact with the doctor is very important, sometimes it’s not so much the question of clarifying their doubts, isn’t it!? But this question of being able to, able to close isn’t it!? Being able to close the process somehow, I think it makes a lot of difference*.” (Psy2) | All fertility specialists indicated that support after unsuccessful treatment should facilitate patients’ grieving process: help patients to identify alternative fulfilment life goals and/or paths (e.g., treatment with donated gametes/embryos, adoption, foster family, childfree lifestyle) beyond biological parenthood, recognise and focus on the positive things they have in life and that result from treatment, accept their inability to have biological children and/or manage social interactions related with parenthood.  “*Indeed, the inability towards failure and the impotence of obtaining what they [patients] have sought us for and what others have and they will not have, and therefore, acceptance for me here I think is, is what many patients lack*.” (Fs5)  “*in the end, it’s helping them to see what is good about them and the projects they can develop together*.” (Fs1)  “*I think this intervention has to show that in life people have to have several interests, because unfortunately people do not always succeed in all areas, but they [patients]) have to focus on family, friends, activities that people like to do, don’t they!? at work, and so there’s actually other things besides that*.” (Fs1)  GYN/OBS referred they have a medical appointment to give the beta hCG test result and answer patients’ questions and concerns. One GYN/OBS added that in their clinic, there is an additional short-term medical appointment after unsuccessful treatment for patients to ask questions and clarify their process. This appointment is well-accepted by patients and perceived by the GYN/OBS as beneficial to facilitate their grieving process. Indeed, patients have several questions after unsuccessful treatment, although sometimes it is difficult for GYN/OBS to have in-depth scientific explanations for their unsuccessful treatment.  “*But I still think, as director of the [fertility] clinic, that this offer [short-term medical appointment after unsuccessful treatment] is necessary (…) From what I can say from my personal experience is that, indeed, they [patients] leave the clinic in a much more comfortable and peaceful way, I have no doubts, over the years that I have accompanied these couples, this is crucial*.” (Fs4) |
| **Category: High fit between *Beyond Fertility* and expressed patients’ and HCPs’ needs** | Patients referred that *Beyond Fertility* is an adequate name (with only one patient suggesting *Beyond Infertility* as an additional acceptable alternative). It conveys a sense of help and the idea of finding other things in patients’ life beyond their fertility path. Overall, patients perceived that the *Beyond Fertility* logical model was holistic and tailored to their needs. Patients appreciated its format, including individual/couple and group sessions, particularly the later (considering its previously mentioned benefits), and six patients agreed that including both formats would allow working on different goals.  “*yes, the possibility of a help, we don’t know very well what this help will be, we don’t know very well how it will be, how it will help us, but maybe I, I look at this help with a little of hope, in the sense that I can understand what’s going on with me*.” (Pa2)  *“to me it almost suggests thinking about strategies or things in our life, discovering things in our life beyond this problem, things that can help us to overcome this because we are not just a woman with [fertility] problems, or a couple [with fertility problems], right!?*” (Pa3)  “*I agree with Pa6, I think both the individual and the group [sessions] are important, because they have different goals, I think, for sure, don’t they!?*” (Pa3) | Psychologists referred that *Beyond Fertility* is an adequate name, conveys a sense of a global approach and the idea of finding and pursuing a new lifestyle beyond patients’ fertility path. Overall, psychologists perceived that its logical model was holistic and integrated all the required processes and outcomes. They appreciated its format, including both individual/couple and group sessions, particularly the latter, considering its previously mentioned benefits.  “*yes, even more, because parenting is not infertility, isn’t it!? So here, here, the grief is the grief towards fertility, not parenthood, right!? Because parenthood can be built beyond fertility, right!?*” (Psy2)  “*Regarding the name, it seems fine to me because somehow it is comprehensive and introduces the issue of infertility and the issue beyond fertility and allows you to encompass a lot and, therefore, allows for several approaches and several perspectives*.” (Psy1)  “*I think the group intervention, I think it can make a lot of sense*.” (Psy1) | Fertility specialists referred that *Beyond Fertility* is an adequate name, conveys a sense of finding other things beyond parenthood. Overall, fertility specialists perceived its logical model was holistic and integrated all the required processes and outcomes. They appreciated its format, including both individual/couple and group sessions, particularly the latter, considering its previously mentioned benefits.  “*indeed, the balance is achieved precisely with these four (Beyond Fertility mechanisms of change) [laughs]. If all are achieved, we get there. Effectively achieving the balance is what is most difficult. In my opinion, these are the essential ones, and this is the way*.” (Fs5)  “*Yes, it seems to me these four areas cover all that, in the end, all the support we want to give the couples at this stage, right!?*” (Fs6) |
| **Theme: Challenges in implementation at fertility clinics** | | | |
| **Category: High willingness to engage with *Beyond Fertility*, but HCPs have concerns about patients’ engagement** | All patients were willing to engage with *Beyond Fertility*. Four patients highlighted that patients’ willingness to receive support might depend on their individual characteristics, particularly for the group sessions. Indeed, two patients referred that their male partner would be less willing to engage with *Beyond Fertility*, as they are more reserved and/or believe they have their own strategies to cope with the treatment burden.  Everyone: “*Yes (without a doubt/completely) [willing to engage with Beyond Fertility]*.”  “*I, I think, I think all couples would somehow be receptive to a professional approach, so from psychology, to understand if they really, if they need to have a support or not*.” (Pa6)  *“But maybe other people think differently and don’t feel comfortable being among strangers*.” (Pa12) | All psychologists were willing to promote *Beyond Fertility*. Psychologists agreed that patients’ willingness to engage with *Beyond Fertility* might depend on their individual characteristics, referring that woman might engage, still it would be challenging to have the male partner engaged. One psychologist stressed that based on their experience, most fertility couples would not be willing to receive psychosocial support in a group format.  “*couples’ adherence to group interventions in XXX [their fertility clinic] I never had much acceptance, I have been asking couples that, I use to ask them, I don’t know if it’s a cultural issue (…) couples do not feel very comfortable with the situation of the group intervention*.” (Psy1)  “*another thing is to share with a couple who is going through the same experience, and I think that most of them will not be all, but most of them this aspect of sharing with someone who is going through the same process is therapeutic and is beneficial*.” (Psy2)  “*we can even have women engaging. It is more difficult to involve the couple, the male element to come. Even in this component of the group, of sharing, of being with other people, we will have female elements that will engage well, and then we will have males that will not*.” (Psy2) | All fertility specialists were willing to promote *Beyond Fertility*. All agreed that patients’ willingness to engage with *Beyond Fertility* would depend on patients’ individual characteristics. Four fertility specialists referred that some patients might not feel at ease sharing their personal experiences in the group, and one GYN/OBS highlighted that it might be challenging to maintain patients’ engagement over time (in particular the male partner).  “*But this is such an intimate matter that sometimes people may not want to be sharing these intimacies with strangers*.” (Fs1)  “*And, and so it is, there are actually couples who are very introspective, very closed and who are definitely going to refuse group therapy so to speak (…) but each case is unique*.”(Fs3)  “*we’re going to have some difficulty in getting couples to keep up with all the sessions*.” (Fs6) |
| **Category: Patients’ barriers: impact on daily work routine and financial costs** | In-person psychosocial sessions imply work absences (which patients have several due to the treatment medical procedures), travel costs and time (in particular for those from distant locations), need to present justifications at work, and associated visibility and stigma. Indeed, one patient also referred that even if they had been offered psychosocial support, they would have only engaged if they were scheduled for the same day of a medical appointment. The patient receiving psychosocial support in the private sector referred it is financially costing.  “*but there are couples, I’ve met couples from XXX [from the Portuguese archipelagos]. They don’t come to XXX [fertility clinic] for a [psychosocial] appointment, I would say.*” (Pa7)  “*It’s just that I should not feel shame, should I!? But that’s what I feel sometimes, honestly. Apart from feeling very exposed. One thing is to say: look, I will be absent [from work], I have a medical appointment. And they don’t even ask me [why] (...) but the documentation goes through several hands, and the fact that it has reproductive medicine there.*” (Pa4) *-“Even more if it says psychology of reproductive medicine.*” (Pa8) | In-person psychosocial sessions imply work absences for patients (which they already have several due to the treatment medical procedures), need to present justifications at work, and travel costs (in particular for those from distant locations). The time of the appointments in the clinic is not convenient for patients (although one psychologist have referred that for those patients who are from distant locations, they try to schedule support on days of the patient’s medical appointments). Receiving psychosocial support in the private sector is financially costing for patients. Two psychologists agreed that having in-person sessions in the clinical setting after unsuccessful treatment is not beneficial for patients, as it might trigger negative emotions and thoughts. One psychologist referred that some patients might not have access to technological resources to receive online support.  “*but when they are discharged, when the process ends, right!? It implies coming [patients] to our appointment on purpose, and sometimes we have couples who clearly don’t come because of that (…) they lose many hours to come, lose the whole day of work between coming and going, right!?*” (Psy2)  “*it’s almost an ambivalence (…) being again in a context that everything reminds them the treatments and the process they undergone, and they often want to overcome and want to forget*.” (Psy1) | In-person psychosocial sessions imply work absences for patients (which they already have several due to the treatment medical procedures) and travel costs (in particular for those from distant locations). The appointments time in the clinic is not convenient for patients (with one GYN/OBS stressing that this does not happen in the private sector). However, undergoing fertility treatment in the private sector is financially costing for patients. One nurse referred that having in-person sessions in the clinical setting after unsuccessful treatment is not beneficial for patients, as it might trigger negative emotions and thoughts. One GYN/OBS also referred that some patients might not have access to technological resources to receive online support.  “*because there are many [couples] who, after going through the whole process, find it difficult to enter the clinic. We have this idea, and they also convey that to us*.” (Fs3)  “*Sometimes it’s not even because they don’t consider it [psychosocial support] important, it is due to work issues, they can’t skip work, one [member of the couple] can but the other one can’t, so this is getting more and more difficult, isn’t it!? And they sometimes feel that this is necessary, but it’s complicated, even more because of the time we’ll need to schedule them, isn’t it!?*” (Fs4) |
| **Category: Clinical barriers: complexity in identifying eligible patients and lack of human resources** | In general, the NHS reimburses three cycles of IVF/ICSI. Notwithstanding, the NHS has some additional criteria, for instance, the age limit of 40 years and the Body Mass Index (BMI). Fertility treatment is prolonged in time primarily due to the long waiting lists (up to several years) with uncertainty about access and time of future cycles. Some patients undergo treatment cycles in the private sector during the waiting periods. Two patients described the waiting room as a funeral.  “*It’s being like a time bomb at the bottom, it’s about having to say it like this: -okay, we will reach, we reach 40 [years old], right!? and that’s it, there’s the end*.” (Pa13)  “*I have been waiting for 3 years, and it looks like 30 years*.” (Pa5)  “*I faced three waiting lists, three!*” (Pa12)  “*I’ve been on the waiting list for 2 years, I was called now, because I turned 40 [years old], but I’ve already had many treatments in the private [sector] (...) there are already 6 attempts*.” (Pa4)  “*The waiting room sometimes looks like a wake, doesn’t it!? We are all there, there, it seems that we are waiting, watching over someone. I, I often associate that room with a wake*.” (Pa7) | The NHS reimburses three cycles of IVF/ICSI, although it has some additional criteria - some patients are only offered one or two cycles (e.g., due to age). Fertility treatment is prolonged in time due to the long waiting lists (up to two years) - some patients undergo treatment cycles in the private sector during these periods. After unsuccessful treatment, those patients who are eligible for are offered additional cycles with donated gametes or embryos (the number of cycles reimbursed by the NHS cycles is not yet well-defined), but these cycles have several circumstantial constraints (reduced number of clinics offering it and/or being recently offered, prolonged in time and reduced number of donors). Their fertility clinics receive patients from inland and distant regions, as few public clinics offer second-line treatment. There is an increasing number of patients, lack of HCPs, in particular psychologists - only one psychologist for the whole fertility department, additionally supporting other health departments - lack of time, and lack of physical spaces in the department. One psychologist also mentioned bureaucracy constraints to implement a psychosocial intervention in the clinic.  “*and at that time [after unsuccessful fertility treatment with own gametes],the couple is no longer in the last treatment mode, to open up a little bit, in the end, another window of hope, another window of hope that maybe they can achieve it after all [treatment with donated gametes or embryos].*” (Psy3)  “*it’s the time, at this moment I think it is, at least I speak for myself, it’s the time issue, because I’m not only [working] in the fertility department, so nor only in the fertility support appointment, so I have to respond to gynaecology, to the valences of gynaecology, to the other valences of gynaecology and, therefore, I am not entirely in the fertility department, I am also in other valences that also consume a lot of my time*.” (Psy1) | The NHS reimburses a limited number of cycles of IVF/ICSI and has some additional criteria (for instance, the age limit of 40 years), so some patients may be only offered one cycle. One GYN/OBS referred that the hospital does not offer additional cycles when patients have a severe bad prognosis. Fertility treatment is prolonged in time due to the long waiting lists (up to one year). After unsuccessful treatment, some patients may undergo additional cycles in the private sector, and those who are eligible are offered additional cycles with donated gametes or embryos, although this latter has several circumstantial constraints (e.g., prolonged in time).  “*This is already a totally different level, this is, it can also be a grieving about the possibility of having children with their own gametes, so this is still a last cycle, isn’t it!? that’s not to say that it’s impossible for them to become parents, right!? So this is still a last cycle, buy it may or may not be the last one, because they [patients] may want to opt for a donation later on*.” (Fs1)  “*But then (after unsuccessful treatment) we can offer alternative options, and therefore the alternatives can go through, as already mentioned here, gametes or embryos donation*.” (Fs1)  The hospital receives patients from all parts of the country, as few public hospitals offer second-line treatments. Two GYN/OBS referred that in their clinic, from the moment patients initiate their treatment, the monitoring process by the fertility GYN/OBS is carried out on a rotative schedule. The GYN/OBS and nurses are the HCPs with more contact with patients, and EMB have less. There is an increasing number of patients, lack of HCPS - in particular psychologists - as all support several health departments, lake of time, difficulty in managing dynamics within the health specialities.  “*this is all related to the lack of, of human resources and the, the scheduling time, because the number of doctors decreases, the number of psychologists decreases, but patients do not decrease, on the contrary, they increase, infertility is increasing more and more, they [patients] arrive later and later on, their prognoses are worse, aren’t they!?*” (Fs4) |
| **Theme: Suggestions to improve acceptability and feasibility of *Beyond Fertility*** | | | |
| **Category: Sponsoring and signposting: the whole fertility team should be involved** | All patients agreed the nurses (preferably, with whom they feel a closer relationship) or the fertility doctor could introduce the *Beyond Fertility* and the possibility of being later contacted by the psychologist, as patients have already established a relationship with these fertility specialists. All patients were willing to be directly contacted by the psychologist, but the majority agreed that this prior in-person referral would be important to promote patients’ engagement with the intervention. Two patients also suggested that the *Beyond Fertility* intervention could be included in the paper medical information patients receive at the beginning of their treatment, and three patients also suggested being introduced in the clinic by the psychologist.  “*I think that it should be presented to everyone and then be volunteer, voluntarily or through the contact you have with, mainly with the nurses, isn’t it!? At least I feel there is a link, a stronger connection with the nurses.” (Pa7) -“I agree with, with Pa7. I think the nurses end up giving us more psychological support*.” (Pa1)  “*We’ve been there[in the clinic] for so long between, between [appointments], we’re called for, for an appointment with, for a, for an ultrasound, for an appointment with the doctor, for, for the care of the nurses, because, why not we be also called during these periods at the beginning of the process for an appointment with the psychologist!? (Pa7: I think so too). For the psychologist to have, to have a closer relationship with the doctors and the, and the technicians of, the nurses, the ultrasound technicians*.” (Pa10)  “*psychological appointments are important. And, and I think there is not everyone who, by phone call, will agree to engage with it, this psychological support program, maybe personally has more impact, I would say*.” (Pa10) | Two psychologists agreed the fertility doctor could introduce *Beyond Fertility* and the possibility of being later contacted by the psychologist, as patients have already established a relationship with these fertility specialists. They also referred to the importance of giving patients the contact of the psychologist for them to turn to at any point.  “*because for all intents and purposes it was the doctor who accompanied them, it was the doctor who was always there during their path and, therefore, I think it is also a way for the team and the doctor to be able to say: ok, for us the work is done, but there is still more that you can use, and we offer you this*.” (Psy1) | Two specialists agreed that the fertility GYN/OBS could introduce *Beyond Fertility* and the possibility of being later contacted by the psychologist. Still, they believed *Beyond Fertility* should not be presented as an intervention for those patients who will face an unsuccessful treatment but as a support to help them cope with the burden that the last treatment cycle entails.  “*So this program has to be presented not as a program for couples who are going to have a failure, otherwise it seems like we’re already labelling them (…) We can offer them the possibility [Beyond Fertility], because we know that they have already undergone two treatments, and this is a treatment that involves great anxiety and perhaps because it is the last treatment that is possible to undergo, it generates more [anxiety] for them, and perhaps it would be important for them to have a psychology appointment*.” (Fs1) |
| **Category: Logistic implementation: scheduling the *Beyond Fertility* in-person sessions on days of medical appointments, tailoring the number and format of the sessions to patients’ needs, include online and outside of working hours delivery** | Conducting the sessions online and outside of working hours would be the most perceived beneficial format to conduct *Beyond Fertility* to overcome patients’ expressed barriers, with only one patient referring they would prefer in-person sessions. If conducted in-person, patients would like to have the first session scheduled on the same day of their medical appointments.  “*because in that way (online and outside of working hours) nobody knows where I am, I don’t have to miss work, I don’t have to travel, I don’t have work piled up… All this weighs, doesn’t it!?*” (Pa4)  “*yes, but I think, I think so, or at least it creates greater flexibility [sessions in an online format], at least in my case, if it were otherwise, I would not be able to participate*.” (Pa2)  Patients agreed that the number and format of the *Beyond Fertility* sessions should be tailored to patients’ needs, as patients might need more individual/couple sessions before the group sessions. One couple suggested having a final individual/couple session to evaluate patients’ overall process.  “*I think the psychologist should, should, in the first individual session, evaluate if this would be enough for that particular couple*.” (Pa2)  Four patients suggested conducting the sessions after unsuccessful treatment, every week or two weeks, and three agreed every month would be too much time between sessions. In addition, three patients referred it would not be important whether psychosocial support after unsuccessful is provided in or outside the clinic. | Psychologists referred that conducting the psychosocial sessions in an online format and outside of working hours would facilitate patients’ engagement with *Beyond Fertility* but agreed that conducting it outside of working hours would imply mental-health professionals to work outside of working hours. If conducted in-person, one psychologist stressed that having car-parking and the sessions scheduled on the same day of the patient’s medical appointment would facilitate their engagement. Regarding the number of sessions, one psychologist referred that patients could benefit from more than one individual/couple session before initiating the last cycle. After unsuccessful treatment, one psychologist referred that conducting the sessions outside the hospital could facilitate patients’ engagement. One psychologist also highlighted the importance of having a structured and goal-oriented protocol for the group sessions.  “*we always try to schedule psychology on the day they [patients] come to the hospital for other appointments, right!? In a way to facilitate [patients’ engagement].*” (Psy2)  “*Therefore, I have a period of time to [schedule] appointments, I start working at 8:00 am, and therefore it goes until 3:30 pm. And so, the afternoon period, from which many times couples could benefit from some support [is not possible].*” (Psy1)  “*I think so, I think so, not so violent [receiving support outside the clinic], because it’s returning again to a place that went wrong, that didn’t give them what they wished*.” (Psy1) | Fertility specialists referred that conducting the psychosocial sessions in an online format and outside of working hours would facilitate patients’ engagement with *Beyond Fertility* due to their constraints, but two fertility specialists agreed that conducting psychosocial sessions outside of working hours would imply mental-health professionals to work outside of working hours. One GYN/OBS referred that conducting the sessions in an online format also implies reduced resources from the hospital (i.e., less use of physical spaces and reduced number of professionals and patients in the hospital).  “*the fact that there are many sessions, if they are in-person, I don’t know if couples will easily accept them. I think doing the group sessions outside of working hours, via Zoom, will increase acceptance. Because one thing is going to two in-person appointments, another thing is going to seven appointments, and I don’t know if couples are very receptive to that.*” (Fs1) *-“Yes, yes (…) I think the use of technologies here can be an asset*”(Psy1).  *“virtual means seems to me, even for the management of the space, the department, and the presence of all [in the clinic], it seems to me a way to, to be considered*.” (Fs6)  The format (individual/couples; group) and the number of psychosocial sessions should be tailored to patients’ individual characteristics, as patients might need more individual/couple sessions before the group sessions. One nurse highlighted the need for more than one individual/couple session before patients initiate their last cycle.  After unsuccessful treatment, two fertility specialists referred that conducting the sessions outside the hospital could facilitate patients’ engagement, with one GYN/OBS referring the opposite, claiming that the hospital could be more straightforward as patients are already familiarised with the context.  “*I think, I think there could be an alternative here, of actually choosing between group sessions or individual sessions (...) each time it is more and more proven that the treatment has to be adapted to each couple, in the field of psychology this is still so much more [important], isn’t it!? I think this possibility should be given, this alternative for the couple to choose*.” (Fs4)  “*I mean, it’s like that, it’s after work for couples, but in fact for the person, for the people delivering the intervention end up working outside of working hours*.” (Fs1) |
| *Note*. HCPs = healthcare professionals; Pa = Patients; Psy = Psychologist; Fs = Fertility specialist; GYN/OB = obstetrician and gynaecologist; EMB = Embryologist | | | |
